# Assessing greenhouse gas emissions in a primary care subdistrict in Cederberg, South Africa

**DOI:** 10.1186/s12913-025-13489-9

**Published:** 2025-10-22

**Authors:** Patricia Nayna Schwerdtle, Claudia Quitmann, Alina Herrmann, Sanskrithi Thakur, Claire Adams, Kiran Jobanputra, Robert Mash

**Affiliations:** 1 Heidelberg Institute of Global Health, Medical Faculty Heidelberg University, Heidelberg University Hospital, Heidelberg, Germany; 2 Division of Family Medicine and Primary Care, Stellenbosch University, Cape Town, South Africa; 3 Health Care Without Harm, New Delhi, India; 4 Cederberg Sub-District, West Coast District, Rural West, Western Cape, South Africa; 5 Climate Action Accelerator, Geneva, Switzerland; 6 Institute of General Medicine, Medical Faculty University of Cologne, University Hospital Cologne, Cologne, Germany

**Keywords:** Climate change, Mitigation, Carbon footprint, Primary health care, Environmental sustainability

## Abstract

**Background:**

Climate change poses a significant threat to global health, risking decades of progress in public health gains and threatening the ability of developing countries to achieve universal health care goals. In response, healthcare systems worldwide are beginning to assess and mitigate their environmental impacts, ideally alongside building their resilience to climate change. Most of this work has centred on high-emitting health systems in high-income countries, yet a large number of developing countries are also committed to reducing the environmental impact of health care and in doing so pursuing a clean development trajectory. This study evaluates the emissions of a primary healthcare network in the Cederberg subdistrict, South Africa, using a standardized greenhouse gas accounting tool. The findings aim to inform mitigation strategies for Cederberg and may offer insights to other resource-constrained settings.

**Methods:**

A hybrid approach, combining bottom-up and top-down calculations, was employed to quantify greenhouse gas emissions across Scopes 1, 2, and 3 of the Greenhouse Gas Protocol, using the Healthcare Without Harm Climate Impact Check-up Tool. Key operational areas included energy use, transportation, waste management, procurement, and water use. Activity data were collected for 2023 through an iterative process of reviewing facility records, interviewing staff, and direct observations involving facility visits.

**Findings:**

The emissions for the Cederberg primary care subdistrict, including six clinics (two large and four small) totalled 1,228 tonnes of carbon dioxide equivalent (CO₂e) per annum, which equates to 10.5 kg CO₂e per consultation. Of these emissions, 51% were generated by the extra supply chain (mainly pharmaceuticals and medical instruments and equipment), inhalers (34%), purchased electricity (8%), transportation – employee commuting (4%), stationary combustion (1%), mobile combustion (1%), electricity transmission and distribution losses (0.5%) and waste incineration (0.5%).

**Conclusion:**

This study provides the first published GHG emissions assessment for primary care in South Africa. The findings emphasize the importance of reducing emissions while maintaining essential services and advancing universal health coverage, with decarbonization offering potential environmental, economic, health, and social co-benefits. This work supports global efforts to decouple health outcomes from environmental harm and lays the groundwork for climate-resilient, low-carbon healthcare in similar settings.

**Supplementary Information:**

The online version contains supplementary material available at 10.1186/s12913-025-13489-9.

## Background

### Climate change and health system challenges

Health systems globally are under growing pressure to reduce their environmental footprint while simultaneously adapting to the escalating health risks posed by climate change. This dual challenge is particularly acute for under-resourced systems in regions most vulnerable to climate impacts. In response, 84 countries have committed to building climate-resilient health systems, 77 to low-carbon healthcare, and 43 to net-zero targets under the COP26 Health Program, led by the World Health Organization (WHO) [1]. Despite these commitments, implementation progress remains slow [2, 3].

### National context - South Africa

Although South Africa has not formally endorsed the COP26 Health Program, both national and provincial governments have made climate pledges. The Western Cape Province, in particular, has committed to net-zero emissions by 2050 [4]. Within this policy framework, the provincial Department of Health and Wellness has committed to reduce carbon emissions by 30% by 2030 and is responsible for implementing health sector-specific strategies to meet the Provincial goal.

South Africa contributes approximately 1.2% of global GHG emissions [5], placing it among the world’s top 15 emitters. This status is largely due to its dependence on coal-based electricity and an energy-intensive economy with limited uptake of low-carbon alternatives. The energy sector alone accounts for approximately 85% of the country’s total emissions, with notable contributions from energy industries and transport [6]. These figures highlight the central role of energy systems in shaping South Africa’s emissions profile and the urgent need for a transition to cleaner energy sources.

### Global healthcare emissions landscape

The healthcare sector accounts for roughly 4.6% of global GHG emissions, amounting to 54.4 gigatonnes from 189 countries in 2015 [2]. High-income countries contribute disproportionately: the United States, China, and the European Union collectively account for 56% of global healthcare emissions [7]. On a per capita basis, countries such as Canada, the United States, Switzerland, and Australia exceed 1 tonne of CO₂e per person, compared to the global average of 0.28 tonnes [7].

In 2021, 91% of healthcare-related emissions originated from countries with very high Human Development Index (HDI) scores. Emissions per person in these countries were 2.6 times higher than in middle-income countries and 8.4 times higher than in low-income countries. Evidence suggests that increases in CO₂e emissions correlate with improvements in health outcomes only up to a threshold of 400 kg CO₂e per capita annually. Beyond this point, higher emissions do not translate into better outcomes, indicating that health systems can deliver quality care without excessive emissions. Achieving net-zero healthcare emissions while maintaining or improving health outcomes will require improvements across facilities, energy systems, operations, supply chains, and care delivery [8].

Emerging evidence indicates that healthcare in Low and Middle Income Countries (LMICs) is more carbon intensive per unit of expenditure than in high-income countries (HICs) [9]. As these systems expand to meet Universal Health Coverage (UHC) targets, emissions could rise substantially - potentially increasing the global healthcare carbon footprint by over 16% [9]. Unlike some high-income countries, such as England, where emissions have decoupled from growth, healthcare emissions in most LMICs continue to rise with service expansion. This highlights the urgent need for context-specific, low-carbon development strategies that align emissions reduction with improved health access [10].

### Climate change and health in South Africa

South Africa is experiencing the effects of climate change, with national average temperatures having increased at twice the global rate since 1990. The country is facing more frequent extreme weather events, including heatwaves, prolonged dry spells, and more intense rainfall [6]. These changes are undermining water security through more frequent droughts and widespread shortages. The health system is likely to come under increasing strain as a result, due to higher disease burdens, infrastructure damage, supply chain disruptions, and pressure on emergency and clinical services [6].

### State of evidence on healthcare emissions in LMICs

GHG assessments of healthcare systems have been reported in high-income settings across Europe (England, Scotland, Austria, the Netherlands), North America (Canada, USA), Oceania (Australia), and Asia (China, Japan). Multi-country analyses using global datasets have also been published [9]. However, there is a critical gap in data from LMICs, particularly in Africa. Two studies from Nigeria highlight healthcare’s contribution to climate change. Sumayya et al. (2023) used primary data from five hospitals to estimate GHG emissions, identifying electricity use and medical waste as major sources [11]. One study in Nigeria applied time-series modelling (2006–2020) and found that public health spending significantly increases national carbon emissions [12]. Another study in South Africa estimated emissions from a government health department vehicle fleet in South Africa and proposed mitigation strategies [13]. Another Nigerian study assessed emission reductions through energy-efficient equipment [14] as one way forward. Baddley and Rasheed (2023) evaluated healthcare emissions across 11 settings in eight LMICs, including primary, secondary, tertiary, and quaternary facilities, but argue that more research on evaluation of carbon footprints of heath care providers in LMICS are needed. Together, these studies highlight the need for both facility-level and system-level decarbonisation strategies in LMICs. To our knowledge, our study is the first to estimate the GHG emissions of a primary care network in South-Africa.

### Importance of emissions tracking in LMIC health systems

While health systems in LMICs are often assumed to contribute minimally to global emissions [15, 16], this assumption warrants ongoing verification. Demographic growth, urbanisation, and the pursuit of UHC are expected to increase healthcare-related emissions. Systematic GHG assessments in LMICs can guide climate-resilient and sustainable development. Ideally, health system strengthening should be decoupled from emissions growth [17]. Avoiding a high-emissions development path brings multiple benefits, including reduced fossil fuel dependency, lower operating costs, improved energy security, and access to sustainability-linked funding. Such a path also promotes healthier environments and advances health and climate goals simultaneously [3, 17].

The objective of this study was to estimate the GHG emissions of a primary care subdistrict in South Africa. Specifically, we aimed to establish a baseline for identifying key emissions hotspots, comparing results to international benchmarks, and analysing emissions by operational domain (e.g., energy, transport, procurement, and waste). The findings will support evidence-based recommendations for reducing healthcare emissions while improving care quality.

## Methods

### Study design

This study assessed the 2023 GHG emissions of public primary care facilities in the Cederberg subdistrict, Western Cape, South Africa, using the Climate Impact Check-up Tool (version 3.3, May 2022: See Supplementary Material) developed by Health Care Without Harm (HCWH) [18]. This assessment was part of a broader initiative evaluating the climate vulnerability and adaptive capacity of the same facilities, supported by the Climate Action Accelerator. Ethics approval was granted by the Health Research Ethics Committee at Stellenbosch University (N23/04/041). Informed consent to participate was obtained from all study participants.

### Study population and data sources

All six fixed, full-time public primary care clinics in the Cederberg subdistrict were included. No sampling was required, as the goal was to account for emissions from the entire subdistrict primary care system. Quantitative secondary data were obtained from subdistrict managers, including aggregated activity data from facility records and administrative systems. Where necessary, additional data were collected directly at the facility level. Site visits and direct observations by the research team helped verify facility infrastructure and operations, and determine the presence or absence of specific emission sources (e.g., anaesthetic gases or diesel generators), ensuring alignment with the Check-up Tool’s categories.

### Study setting

**Fig. 1:**
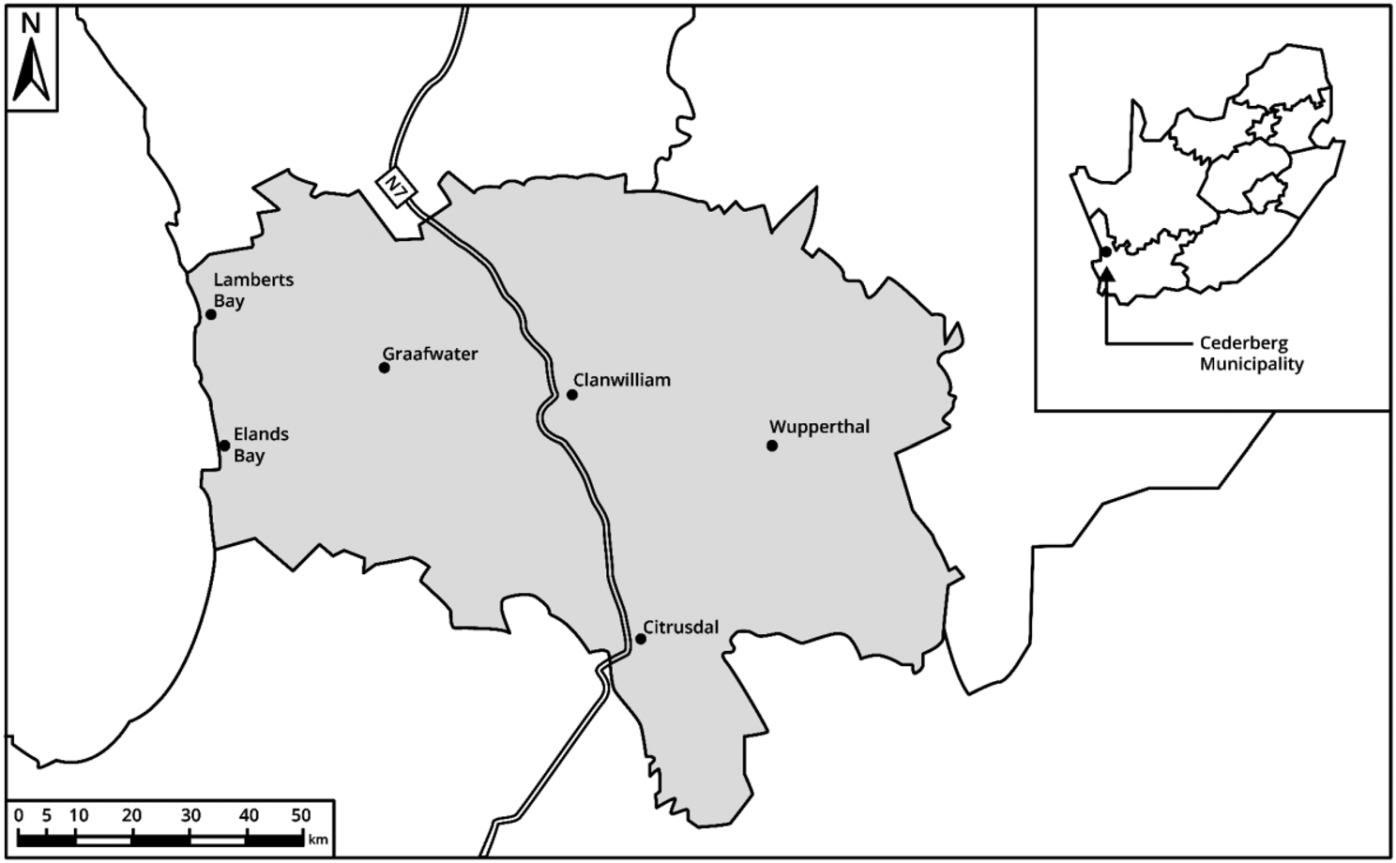
Map of the Cederberg subdistrict and location in South Africa

The Cederberg subdistrict (Fig. 1) is identified as one of the most climate-vulnerable areas in the Western Cape [19], and serves a population of approximately 64,850 − 6.1% aged over 65 and 22.3% under 15 years. Healthcare services included six governmental primary care facilities that were assessed in this study (Table 1) as well as two small district hospitals, one part-time clinic, and five mobile clinics. Rural residents constituted 44% of the population. In 2023, the six clinics conducted 116,585 patient consultations providing preventative, promotive and curative ambulatory primary care services. The clinics employed 58 staff members in total and were nurse-led, supported by clinical and administrative personnel. Patients mainly accessed the clinics on foot but also used public transport and car-pooling. Further information on the six clinics included can be found in Table 1.

**Table 1:** Health facility description

Facility	Number of buildings (consulting rooms)	Shade areas for waiting outside	On site fuel use (diesel generator)	Electricity storage (inverter and battery)	Mains electricity supply	Solar or renewable energy	Multiple AC window units	Water heating via electrical geyser	Incineration of medical waste off-site	Composting
Wupperthal	1 (2)	Yes	No	Yes	Yes	No	Yes	Yes	Yes	No
Clanwilliam	2 (5)	Insufficient	Yes	No	Yes	No	Yes	Broken	Yes	No
Graafwater	1 (3)	Yes	No	Yes	Yes	No	Yes	Yes	Yes	No
Lamberts Bay	1 (8)	Yes	No	Yes	Yes	No	Yes	Yes	Yes	No
Elands Bay	1 (4)	Yes	No	Yes	Yes	No	Yes	Broken	Yes	No
Citrusdal	1 (8)	Insufficient	Yes	No	Yes	No	Yes	Yes	Yes	No

### Greenhouse gas emissions assessment

Greenhouse gas assessments typically use either bottom-up (activity-based) or top-down (spend-based) approaches. Bottom-up methods rely on detailed data from specific activities or processes within an organisation, offering high precision but requiring substantial data collection. In contrast, top-down methods use aggregated financial or sector-level data to estimate emissions, providing broader coverage with less granularity. In healthcare, combining these methods - a hybrid approach - is often necessary due to data limitations and can improve both accuracy and completeness [20].

### Data collection

In this study, GHG emissions were assessed over a 12-month period (June 2023–May 2024) using the (HCWH) Climate Impact Checkup tool. This tool adopts a hybrid methodology, integrating both activity-based and spend-based data, and aligns with the Greenhouse Gas Protocol [18]. It covers 11 supply chain categories and includes emission factors for 43 countries, along with global averages for other countries. Users are guided through input modules that generate emissions profiles by domain (e.g., energy, travel, procurement), scope, and time, facilitating internal benchmarking and progress tracking.

Data were collected through site visits, staff interviews, and secondary sources, including routine data from the primary healthcare network. Emissions were calculated across Scope 1 (direct emissions from facilities), Scope 2 (indirect emissions from purchased energy), and Scope 3 (other indirect emissions such as procurement and commuting). Table 2 defines the three scope emission areas (GHG Protocol) and the operational areas assessed in the primary care network. While not designed for direct comparison across facilities using different tools, this approach enables healthcare providers to establish credible emissions baselines and improve reporting over time.

**Table 2:** Emission scope descriptions in climate impact check-up tool

Scope	Category
Scope 1 Direct emissions	Stationary combustion: Gas/ diesel oil [litres] (i.e. – Diesel generators) Mobile combustion: Gas/ diesel oil [litres] (i.e. – Patient outreach vehicles)
Scope 2 Energy purchased	Purchased electricity from the grid [kWh]
Scope 3 Indirect emissions	Employee commuting by private car (diesel/petrol) based on distance per year [km] Inhalers dispensed or prescribed [no. of metered-dose and dry-powder inhaler] Electricity transmission and distribution losses of grid electricity [kWh] Incineration of waste [kg] - Municipal solid waste - Clinical mix (unsegregated - biohazardous and hazardous) - Hazardous (Pharmaceutical and hazardous chemicals) ‘Extra Supply chain’ based on spending [South African rand] - Business services - Construction - Information and communication technologies - Medical instruments/ equipment - Other manufactured products - Paper products - Pharmaceuticals

### Data analysis

The Climate Impact Check-up Tool includes an integrated emissions calculator that converts activity-based and expenditure data into carbon dioxide equivalents (CO₂e) using standardised emission factors. GHGs covered under the Kyoto Protocol were included. Country-specific emission factors were applied when available; for electricity, South Africa’s national factor was used–the highest among G20 nations - which significantly influenced the results [19]. Emission factors were drawn from the 2006 Intergovernmental Panel on Climate Change (IPCC) Guidelines, the World Resources Institute/GHG Protocol database, and the UK DEFRA dataset [21–23].

Scope 3 emissions—typically the largest share of healthcare emissions—are the most complex to quantify due to their indirect nature and reliance on supply chain activities beyond the direct control of facilities [24]. The tool employs a hybrid methodology based on the GHG Protocol Scope 3 categories and the World Input-Output Database (WIOD). It calculates emissions for inhalers, electricity transmission and distribution losses, waste, and employee commuting using activity-based data. Remaining supply chain emissions are estimated through a spend-based approach, where users input annual expenditure data by category. These are then matched with emission factors expressed in kg CO₂e per unit currency, as derived from WIOD [25].

This spend-based method facilitates easier emissions estimation, as financial data are often more readily available than mass or volume-based activity data. The WIOD includes 15 procurement categories: business services, construction, food and catering, information and communication technologies, manufactured fuels, chemicals, and gases, medical instruments/equipment, other manufactured products, paper products, pharmaceuticals, water and sanitation, other procurement, electricity, fossil fuels, waste products and recycling, transport. Of these, the final four categories - electricity, fossil fuels, waste, and transport - were excluded from the supply chain estimation, as they were already captured under Scope 1, 2, or other Scope 3 calculations.

## Results

**Fig. 2:**
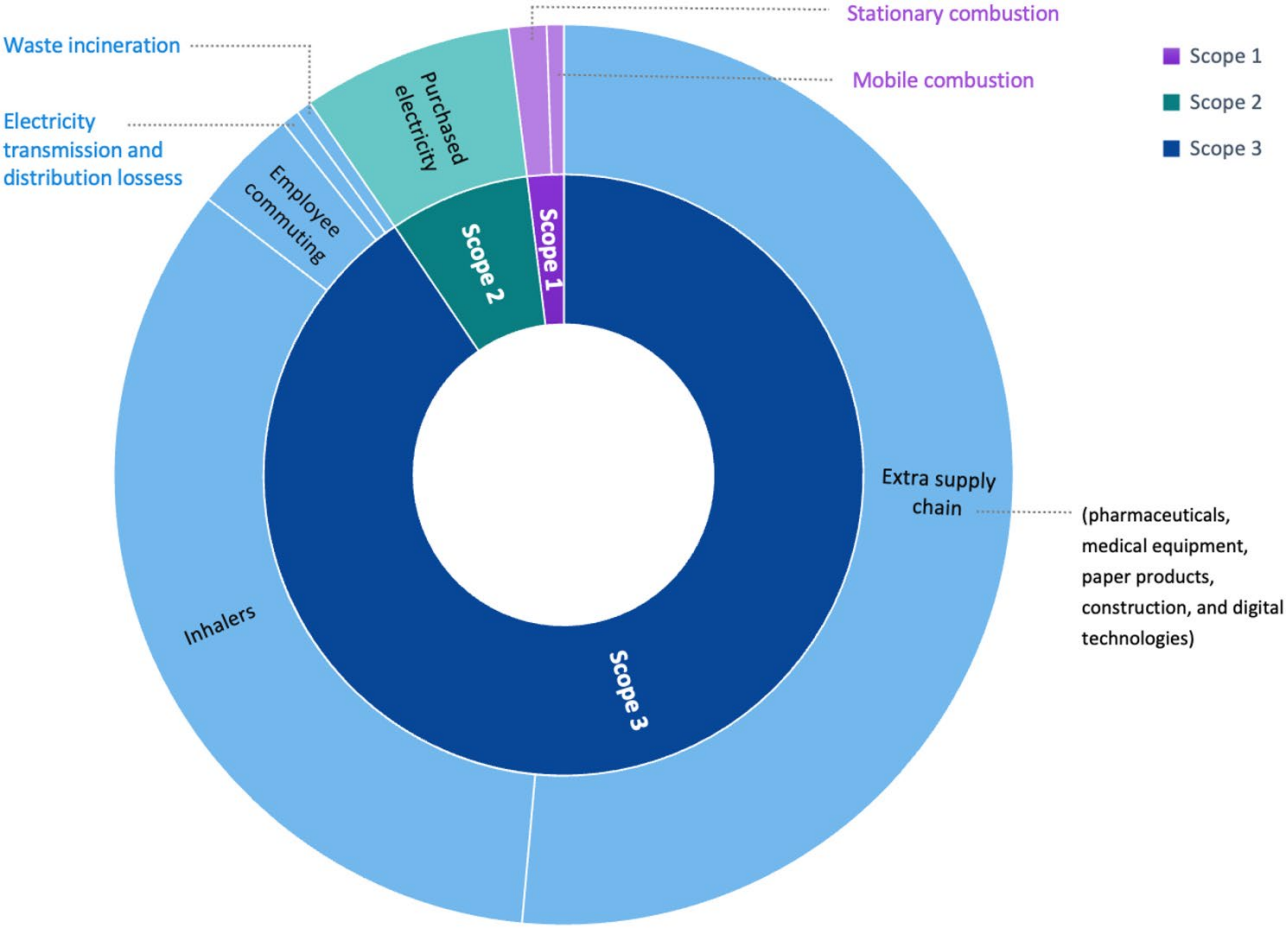
Sunburst diagram of the Cederberg emissions calculation

**Fig. 3:**
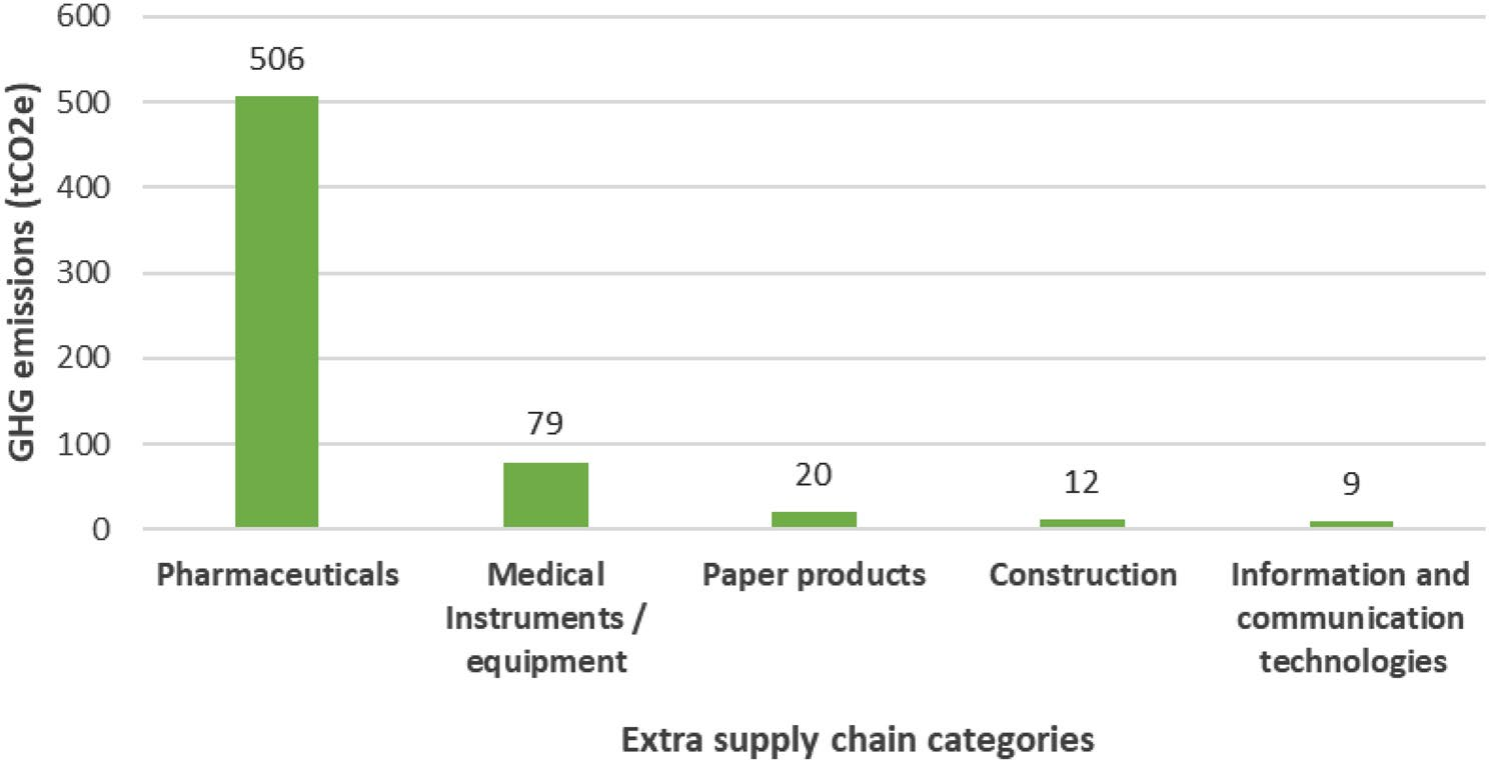
Extra supply chain hotspots - Top 5 emission categories (t CO_2_e)

The total annual emissions were 1,228 tonnes CO_2_e for the primary care facilities in the Cederberg subdistrict, which is 10.5 kg CO_2_e per consultation. Scope 3 contributed 90.5%, while Scope 1 and 2 were only responsible for 1.9% and 7.6%. The contributing factors towards the GHG emissions were extra supply chain (51.5%), inhalers (34.0%), purchased electricity (7.6%), employee commuting (3.8%), stationary combustion (1.3%), mobile combustion (0.6%), electricity transmission and distribution loss (0.6%) and waste incineration (0.6%). The results are summarised in Table 3 and visualised in Figs. 2 and 3.

**Table 3:** Emissions summary table for the Cederberg subdistrict

Scope	Emissions domain	Tonnes of CO_2_e	% Contribution
Scope 1	Stationary combustion	16.17	1.3%
	Mobile combustion	7.62	0.6%
Scope 2	Purchase of electricity	93.37	7.6%
Scope 3	Extra supply chain	632.32	51.5%
	Employee commuting	46.25	3.8%
	Inhalers	417.77	34.0%
	Electricity transmission and distribution losses	7.84	0.6%
	Waste incineration	6.84	0.6%
Total		1228.18	100%

### Scope 1 - Direct emissions

- **Stationary combustion** represents emissions from on-site fuel combustion for heating, hot water, or other stationary energy uses within healthcare facilities. All clinics have electric geysers (rather than gas boilers) and stationary combustion was attributed to diesel generators, which were used when the grid goes down. Diesel generators contributed 16.17 tCO_2_e (1.3%) to the total emissions.
- **Mobile combustion** included emissions from fuel used in vehicles owned or leased by healthcare facilities, such as ambulances, delivery trucks, or fleet vehicles. In Cederberg, the fuel needed for mobile health clinics that provided outreach services resulting in 7.62 tCO_2_e (0.6%) of the total emissions.

### Scope 2 - Purchased electricity

- **Purchased electricity** covers emissions from the electricity consumed by healthcare facilities, including emissions from the generation of electricity sourced from the grid. The electricity consumption from the six facilities generated 93.37 tCO_2_e (7.6%) and 101.2 tCO_2_e in total if we consider the electricity transmission and distribution losses (measured in scope 3).

### Scope 3 - Indirect emissions - supply chain

- **Extra supply chain.** Scope 3 emissions were separated into supply chain and extra supply chain. These were GHG emissions from goods and services purchased by healthcare facilities that went beyond core medical supplies (such as office equipment, furniture, and construction materials) and included pharmaceuticals, business services, food and catering, information and communication technologies, medical instruments/ equipment, paper products, water and sanitation, and other procurement. This category generated 632.32 tCO_2_e (51.5%) and were therefore the major contributor to the footprint.
- **Inhalers** included GHG emissions from the production, use, and disposal of inhalers, MDIs (Metered Dose Inhalers) and DPIs (Dry Powder Inhalers). At the time of data collection, only MDIs were prescribed, and no DPIs were in use during the study period. Inhalers contributed 417.77 tCO_2_e (34%) to the annual emissions of the subdistrict, especially MDI with propellants like hydrofluorocarbons (HFCs), which are potent GHG.
- **Employee commuting** measured GHG emissions from healthcare staff travelling to and from work. These Scope 3 emissions depended on distance and mode of transport. Most staff lived within 2–5 km of their healthcare facilities, but a few travelled longer distances, such as 122 km roundtrip for outreach or 45 km for regular work. The employee commute generated 46.25 tCO_2_e (3.8%).
- **Waste incineration** accounted for emissions from the burning of healthcare waste, including both medical and general waste. This process releases carbon dioxide and other pollutants, depending on the waste composition and incineration efficiency. The facilities’ waste was incinerated and comprised of municipal solid waste, clinical mix and hazardous waste, amounting to 6.84 tCO_2_e (0.6%).
- **Electricity transmission and distribution loss** captured GHG emissions associated with energy lost during the transmission and distribution of electricity from power plants to healthcare facilities. This is often overlooked but contributed to the overall GHG emissions with 6.84 tCO_2_e (0.6%).

Figure 3 breaks down the GHG emissions from the extra supply chain, which contributed just over half of all emissions (51.5%). Pharmaceuticals, (medicines, vaccines, contraceptives), medical instruments and equipment (such as surgical instruments), and paper products (such as stationery) formed the top 3 emission sources in the extra supply chain. Pharmaceuticals contributed the highest share with 506.37 tCO_2_e (80.1%). Medical instruments and equipment came next with 79.14 tCO_2_e (12.5%), then paper products 20.16 tCO_2_e (3.2%), construction materials 11.93 tCO_2_e (1.9%), and digital technologies 9.08 tCO_2_e (1.4%). Business services contributed 4.67 tCO_2_e (0.7%) and other manufactured products (including medical consumables) contributed 0.97 tCO_2_e (0.2%).

## Discussion

### Summary of key findings

This GHG assessment found that six fixed, full-time primary care clinics in South Africa’s Cederberg subdistrict emitted a total of 1,228 tonnes CO₂e annually, equating to 10.5 kg CO₂e per consultation. The majority of emissions originated in Scope 3, with the extra supply chain accounting for 51%, primarily due to pharmaceuticals, medical instruments/equipment, and paper products (see Fig. 3). Inhalers contributed a further 34%, followed by smaller contributions from employee commuting (4%), waste incineration, and electricity transmission losses. Scope 2 emissions - purchased electricity - accounted for 8%, and Scope 1 emissions from stationary and mobile combustion were minimal.

### Comparison with other studies

Comparable GHG assessments in primary care settings from African countries and other LMICs are limited. However, in a study conducted in France, primary care emissions were estimated at 1.5 kg CO₂e per consultation, mainly due to patient and physician transport (83%), which was not included in our analysis. A Swiss study estimated 4.8 kg CO₂e per consultation, also dominated by transport and heating. Neither study accounted for emissions from inhalers or medications - two of the largest contributors in our setting. A broader top-down NHS England analysis reported 66 kg CO₂e per consultation; however, differences in scope and methodology limit comparability and highlight the need for transparency and consistency in emissions reporting.

Our findings reflect global trends where Scope 3 emissions dominate healthcare carbon footprints. These emissions arise from the production, transportation, and disposal of goods and services required for care delivery, highlighting the need for context-specific mitigation strategies based on transparent methodologies.

### Supply chains and the LMIC context

In this study, 91% of emissions originated from Scope 3 sources, indicating strong reliance on external supply chains. This is consistent with findings from the Aga Khan Development Network (AKDN), which reported that LMIC healthcare is often more carbon-intensive per dollar than in HICs, due to inefficient procurement, fossil fuel dependency, and weak regulation [9]. In fact, as LMICs expand healthcare access to meet UHC goals, emissions could increase by 382 MtCO₂e annually - a 16% rise globally [26].

Main emission drivers in LMIC healthcare include fossil-fuel-based energy, inefficient transport, and carbon-intensive pharmaceuticals and medical devices. Despite these challenges, decarbonisation (or decoupling health care expansion from emissions) remains feasible using existing tools, methods and technologies. LMICs that produce and export health commodities can also lead supply chain reform.

Currently, few tools support detailed Scope 3 accounting in LMICs. Environmentally Extended Multi-Regional Input-Output (EE-MRIO) models, based on economic data, risk overestimation if not complemented by facility-level or supplier-specific inputs. This supports hybrid approaches that combine top-down and bottom-up data and points to the need for harmonised procurement practices that incentivise supplier decarbonisation.

### Methodological considerations

We used the Climate Impact Check-up Tool, which aligns with the GHG Protocol and enables facility-level input of data on electricity, travel, procurement, and pharmaceuticals. While it effectively tracks emissions and supports mitigation planning, its accuracy depends on the completeness of input data and is not suited to direct comparisons across facilities.

This study relied on available and verified data from subdistrict managers and facility records. Some emissions sources - such as fluorinated gas leakages (cooling gases) leakages, patient commuting, and specific waste streams – were not possible to account for. However, omitting communiting is unlikely to have significantly altered results. given local patterns of transport (e.g., walking, public transport).

### Emissions drivers

A key finding was the substantial impact of MDIs, which use hydrofluorocarbon propellants with global warming potentials up to 3,300 times higher than CO₂ [27]. In this setting, 100% of prescribed inhalers were MDIs. Dry-powder inhalers (DPIs) offer a lower-emission alternative with similar effectiveness and cost [27]. Switching to DPIs could significantly reduce emissions [28] but would require updates to essential drug lists, clinical guidelines [29] and procurement policies.

Building energy use and diesel generators contributed minimally, which aligns with findings from other studies in both LMICs [9] and HICs [20, 30], where Scope 3 sources often account for up to 90% of emissions.

### Study contributions

This is among the first studies to quantify GHG emissions in a primary care setting in Africa. It demonstrates the feasibility of carbon foot-printing in resource constrained settings and highlights the outsized role of supply chain emissions. Notably, this study included medications and inhalers within its system boundaries - rare in primary care research - highlighting their significant contribution to emissions and relevance for mitigation strategies.

While switching to DPIs is already promoted in countries like the UK and Germany [31], such changes require training for health professionals and patients to ensure correct use. There is a need to reduce over-prescription. Antibiotic stewardship, a recognised issue in South African primary care [32], not only reduces antimicrobial resistance and adverse effects but also GHG emissions [33].

Though limited to one subdistrict, this study provides valuable insights for provincial and national mitigation strategies - particularly around procurement and inhaler policy. It offers a replicable method for other African health systems and contributes to closing global data gaps. Given the projected growth in healthcare demand across LMICs, localised studies like this are necessary for developing equitable climate policy and advancing global mitigation.

### Limitations

The assessment was constrained by data availability and completeness. Some emission sources - including cooling gases and business travel - were not measured. While nitrous oxide and anaesthetic gases were not used and hot water was supplied via electric geysers, data on F-gas leakage remain a Scope 1 gap. Waste incineration is conducted off-site; general waste was included, but recycling was minimal at most sites, and mostly involved cardboard recycling. The Check-up Tool was not supplemented with supplier-specific data, and Scope 3 emissions may be under- or overestimated due to the use of top-down methods. Nonetheless, the tool provides a useful baseline and supports capacity-building for future assessments. The hybrid method used is supported by the AKDN and related literature.

### Recommendations

While developing a detailed mitigation strategy was beyond the scope of this study, our findings suggest several general directions for action. The emissions profile - dominated by Scope 3 sources such as pharmaceuticals, inhalers, and medical supplies - points to key intervention areas. Health systems could reduce emissions through greener procurement, supplier engagement in decarbonisation, improved antibiotic stewardship, and transitioning to DPIs where appropriate. Environmentally informed decision aids may support these clinical shifts. Facility-level strategies such as solar energy and efficient lighting should be considered. Following this assessment, waste management improvements are planned including an alternative waste disposal system and in terms of energy – improved insulation and highly reflective roofs.

These suggestions align with recommendations emphasising integrating carbon footprint measurement into UHC, investing in clean energy, and prioritising low-carbon procurement [26]. They also called for a shift toward community-based, preventive, and digital care, and the development of zero-carbon primary care facilities—particularly urgent given the WHO’s projected global need for 378,000 new clinics [26].

## Conclusion

This study provides, to our knowledge, the first published comprehensive estimate of greenhouse gas (GHG) emissions for a primary care subdistrict in South Africa, reporting annual emissions of 1,228 tonnes CO₂e across six clinics in the Cederberg Subdistrict. The findings reveal the predominance of Scope 3 emissions, primarily associated with the supply chain and inhalers, reflecting the sector’s dependence on external production, transportation, and disposal processes.

Direct comparisons of healthcare carbon footprints across countries are constrained by variations in accounting methodologies, tools, data availability, and persistent data gaps. This study highlights the urgent need for comparative research employing consistent accounting approaches across primary care settings in Africa and LMICs to identify differences, support meaningful comparisons, and inform context-specific mitigation strategies.

Healthcare systems in developing countries have a unique opportunity to adopt cleaner development pathways while simultaneously adapting to climate impacts. The Climate Impact Check-up tool proved valuable for tracking emissions. Mitigation strategies targeting green procurement - particularly low-carbon inhalers - alongside energy efficiency, staff behaviours, and waste management, could reduce emissions while generating health, economic, and environmental co-benefits.

## Supplementary Information

Below is the link to the electronic supplementary material.

Supplementary Material 1

## Data Availability

The tools used for this research are open-access and referenced in the manuscript. The data is available upon request from the corresponding author.
