# Supplementary material for: Assessing greenhouse gas emissions in a primary care subdistrict in Cederberg, South Africa
Source: BMC Health Serv Res. 2025 Oct 22;25:1389. doi: 10.1186/s12913-025-13489-9 (PMC12541990; doi:10.1186/s12913-025-13489-9)
Supplement: Supplementary file 1 — Supplementary Material 1 [file 12913_2025_13489_MOESM1_ESM.pdf]

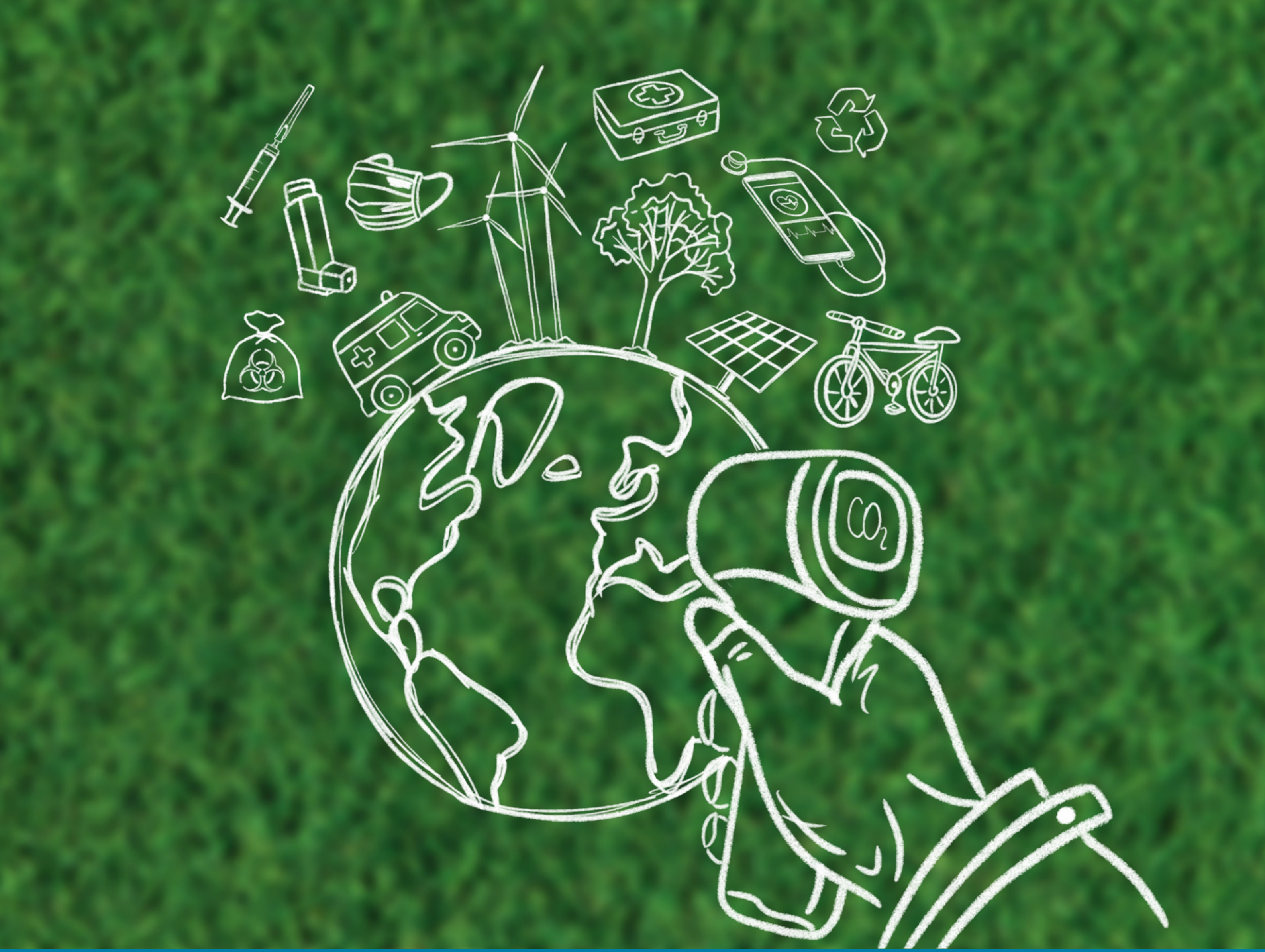

PART 1

How to use Climate Impact Checkup

METHODOLOGICAL GUIDELINES

VERSION 2 - JANUARY 2023

This document is only available for members of the GGHH network.

GLOBAL GREEN and HEALTHY HOSPITALS

Health Care Without Harm

Table of contents

Introduction 6

What does "carbon footprint" mean? 7

What are the greenhouse gases? 8

What are the sources of GHG emissions? 9

How to estimate GHG emissions 11

Principles of GHG accounting 11

Language differences 12

Measurement units in the tool 12

Institutional arrangements 13

What is in the tool 14

How to use the tool 15

User profile 15

How to access the tool 15

Definition of institutional boundaries for emissions estimation 15

Profile information 15

Introduction and general data 16

Notation keys 16

Testing or reporting with Checkup? 17

Scope 1 17

Scope 2 19

Scope 3 20

Extra supply chain 21

Waste 23

Quality checks 25

Results and comparing data 26

Glossary 27

References 29

Annex I – Global warming potentials 30

Annex II – Online platform visualization 34

Annex III - WIOD categories 43

Tables

Table 1 – Summary of the global warming potential values and permanence 8

Table 2 – Scopes 9

Table 3 – Principles of GHG accounting 11

Table 4 – Names of fuels 12

Table 5 – Names of waste streams 12

Table 6 – Sources of GHG included in the tool 14

Table 7 – Notation keys 16

Table 8 – Global warming potentials 30

Table 9 – WIOD categories 43

Figures

Figure 1 – Scopes 10

Figure 2 – Global health care footprint split by scopes 10

Figure 3 – Health care's global emissions by supply chain categories 21

Figure 4 – GHG emissions per scope by different levels of complexity (GGHH LAC members data) 26

Acknowledgments

Authors:

- Antonella Risso, Health Care Without Harm international climate technical and research manager and Climate Impact Checkup project coordinator.
- María Sol Aliano, carbon footprint calculation tool developer and expert consultant.
- Felipe Saavedra, carbon footprint consultant.
- Lucila Citcioglu, Climate technical consultant.

Health Care Without Harm appreciates the collaboration of Global Green and Healthy Hospitals (GGHH) members and the organization's staff in the development and validation of the tool.

- Josh Karliner, Health Care Without Harm international director of program and strategy.
- Jennifer Wang, Health Care Without Harm associate director, international climate program.
- Sonia Roschnik, Health Care Without Harm international climate policy director.

Validation process participants

- Health Care Without Harm staff and partners per organization and location
  Andrea Hurtado Epstein, Health Care Without Harm LAC, Mexico; Anna Fuhrmann, Health Care Without Harm Europe, Belgium; Carol Behne, Climate and Health Alliance, Australia; Carolina Gil Posse, Health Care Without Harm LAC; Clare Westwood, Health Care Without Harm SE Asia, Malaysia; Claudia Lorena Paz Giraldo, Health Care Without Harm LAC, Colombia; Jessica Wolff, Health Care Without Harm US, United States; Keith Edgerton, Health Care Without Harm US, United States; Megha Rathi, Health Care Without Harm Global Team, Switzerland; Mireia Figueras Alsius, Health Care Without Harm Europe, Belgium; Poornima Prabhakaran, Public Health Foundation India, India; Scott Brady, Health Care Without Harm Europe, Scotland; Shweta Narayan, Healthy Energy Initiative, India; Sonia Roschnik, Health Care Without Harm Global team, France; Susan Wilburn, Health Care Without Harm Global Team, United States.

- External experts
  Sally Edwards, PAHO/WHO, Panama
  Factor CO_2 Spain

- GGHH members per institution and location
  Hospital Universitario Austral, Argentina; Hospital Regional Ushuaia, Argentina; Sunshine Coast Hospital and Health Service, Australia; Servicio de Salud de Valdivia, Chile; Department of Occupational Health and Environmental Management, Ministry of Health of Chile (80 hospitals participating); Hospital San Rafael de Pasto, Colombia; Corporación Méderi, Colombia; Clínica Bíblica, Costa Rica; Hospital Calderón Guardia, Costa Rica; Higu Kefale, representing Federal Ethiopia Ministry of Health; Centro Estatal de Vigilancia Epidemiológica y Control de Enfermedades, Mexico; Sunnaas Rehabilitation Hospital, Norway; Radboud University Medical Center, Netherlands; Khoo Teck Puat Hospital, Singapore; Departament de Salut Xàtiva - Ontinyent, Spain; Hospital Lucus Augusti, Spain; Hospital Pedro Hispano, Portugal; Meyer Children's Hospital, Italy; General Hospital of Syros, Greece; Sussex Community NHS Foundation Trust, United Kingdom; Providence Health System, United States; Cleveland Clinic, United States; Ohio State University Wexner Medical Center, United States; Anna-Lisa Mills - Newcastle upon Tyne Hospitals NHS Foundation Trust, United Kingdom.

Copyeditor: Angela Lutz

Design: Paola Dalman

Design and edition coordination: Ana Belluscio, Health Care Without Harm communications team

Abbreviation and terminology

Acronym	Full term
**BAU**	Business as usual
**CH_4**	Methane
**CO_2**	Carbon dioxide
**CO_2e**	Carbon dioxide equivalent
**GHG**	Greenhouse gas
**GPC**	Global protocol for community-scale greenhouse gas emission inventories
**GWP**	Global warming potential
**HCWH**	Health Care Without Harm
**IEA**	International Energy Agency
**INDCs**	Intended nationally determined contributions
**IPCC**	Intergovernmental Panel on Climate Change
**LAC**	Latin America and the Caribbean
**N_2O**	Nitrous oxide
**NDC**	Nationally determined contribution
**SDG**	Sustainable development goals
**UN**	United Nations
**UNEP**	United Nations Environment Programme
**UNFCCC**	United Nations Framework Convention on Climate Change

Introduction

This document serves as a guide to use Climate Impact Checkup's tool for calculating the carbon footprint of health facilities and systems, to support the development of mitigation goals and action plans, and to integrate these actions with other environmental sustainability programs such as sustainable procurement.

Health Care Without Harm's carbon footprinting tool was developed to estimate Greenhouse Gases (GHG) emissions of health care institutions globally by using facility-level data principally from energy consumption, transport, and waste management, as well as other gases relevant to the sector, such as anesthetic gases, cooling gases, and inhalers.

By using Health Care Without Harm's carbon footprinting tool, health care facilities and systems can identify the types and estimate the quantity of their emissions and develop appropriate mitigation plans. Understanding major emissions sources will allow an institution to target their mitigation efforts and maximize results.

By reducing its carbon footprint and moving towards zero greenhouse gas emissions, the health care sector can lead the way forward in this era of climate and environmental crisis and promote a healthy and sustainable future for all[1].

This guideline was divided in 3 documents and parts. See the rest of the guideline parts here.

Context

> *"Climate change threatens to undermine the last 50 years of gains in public health, intensifying heatwaves and extreme weather events, worsening flood and drought, altering the spread of infectious diseases, and exacerbating poverty and mental ill-health. Crucially, the response to climate change brings immense benefits for human health, with cleaner air, healthier diets, and more liveable cities."*
>
> **Lancet Countdown[i]**

Climate change is considered one of the main current threats and challenges of humanity. It generates disturbing changes in the environment and disrupts the delicate balance between our planet's ecosystem and the species that depend on it[1]. Climate action is one of 17 sustainable development goals the United Nations agreed to achieve by 2030, and many of the other goals have a better chance of success if associated with international negotiations approved under UNFCCC.

The health care sector makes intensive use of resources with a current emissions profile estimated at 4.4% of global Greenhouse Gases (GHGs) emissions[2, 3, 4]. As such, it contributes to climate change and the development of respiratory diseases. The health sector, due to its size and influence, can assume a unique leadership role in the fight against climate change[1].

In 2020, Health Care Without Harm participated in the development of WHO guidance for climate-resilient and environmentally sustainable health care facilities. To achieve sustainable and resilient health care facilities, it is necessary -among other things- to understand where greenhouse gas emissions are produced.

[i] Lancet Countdown - https://www.lancetcountdown.org/about-us/

What does carbon footprint mean?

A carbon footprint (also called an inventory) is the sum of greenhouse gas emissions resulting from an activity, the operation of a facility, or the manufacture and use of a product. In this tool, a carbon footprint refers to a quantified list of a facility's GHG emissions and sources[5].

A carbon footprint is also an indicator of sustainability that allows us to identify the contribution of each activity to climate change. Therefore, it is possible to estimate the major sources of emissions and compare the performance of different institutions that carry out similar activities.

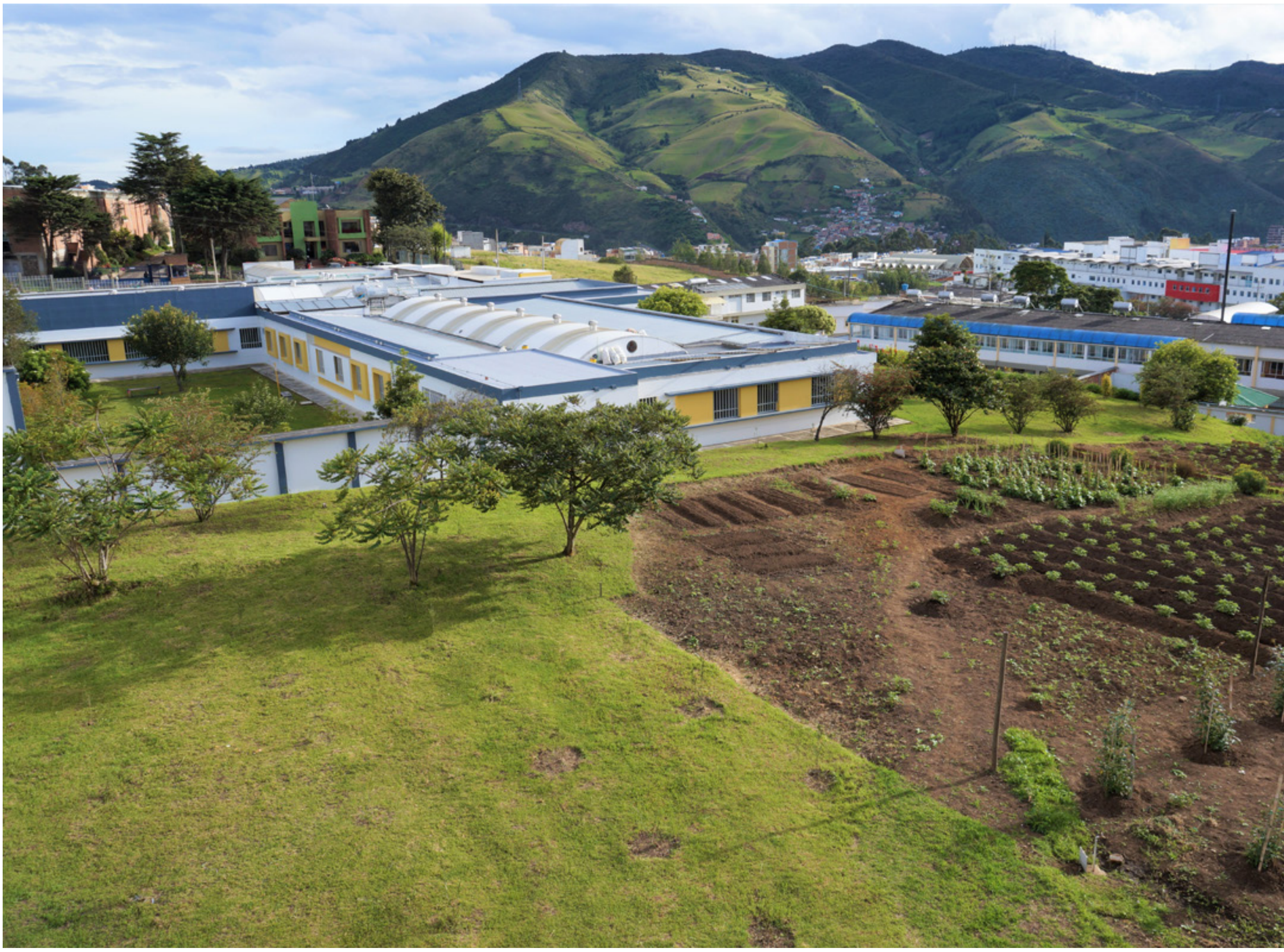

The Hospital San Rafael de Pasto in Colombia uses Health Care Without Harm's tools to calculate its carbon footprint since 2018.
Photo: Hospital San Rafael de Pasto.

What are greenhouse gases?

Greenhouse gases (GHG) are responsible for generating the greenhouse effect in the atmosphere, which can be classified into two types. The first comes from natural causes and makes life possible on Earth by keeping the global average temperature around 15 °C. The second comes from anthropic causes linked to human activities and has as its main consequence an increase in average temperature, which we commonly call global warming.

In addition to carbon dioxide (CO_2), other GHG have different levels of impact in the atmosphere. In order to compare the impact of emissions from different GHG, an equivalence unit called global warming potential (GWP) was defined. This unit converts the emissions of any gas into CO_2 equivalent emissions. For example, if a gas has a GWP of 2, it means that 1kg of emissions of this gas equals 2kg of CO_2.

By using this conversion unit, it is possible to add up and compare emissions of different GHGs in terms of carbon dioxide equivalence (CO_2e).

The following table shows a summary of global warming potential values, while Annex I has a list of all the values used in the tool and their references.

Table 1 – Summary of global warming potential values and permanence in the atmosphere

**Gas**	**Lifetime** (years)	**Global warming potential** (100 years horizon)
Carbon dioxide (CO_2)	Variable *	1
Methane (CH_4)	12	25
Nitrous oxide (N_2O)	114	298
Perfluorinated compounds	740 – 50,000	7,390 – 17,700
Hydrofluorocarbons	1.4 – 270	12 - 14,800
Isoflurane	3.2	510
Sevoflurane	1.8	130
Desflurane	8.9	2,540
R12	100	10,900
R22	12	1,810
R410a	16	16
R600a	12	3

*See IPCC, https://www.ipcc.ch/site/assets/uploads/2018/02/ar4-wg1-chapter2-1.pdf, page 213, note a.
Sources: lifetime of isoflurane, sevoflurane and desflurane[6]; for additional references see Annex I.

What are the sources of GHG emissions?

Causes of climate change are commonly associated with combustion of hydrocarbons. While this globally remains the main cause, there are other sources of GHG emissions, and combustion may occur in many different ways and result from various activities. Therefore, it is necessary to clearly define these sources to avoid double counting while ensuring completeness (which means all possible sources of emissions are considered). To help delineate direct and indirect emissions sources, improve transparency, and provide utility for different organizations, climate policies, and goals, three scopes are defined in the standards[ii] for GHG accounting and reporting purposes[5].

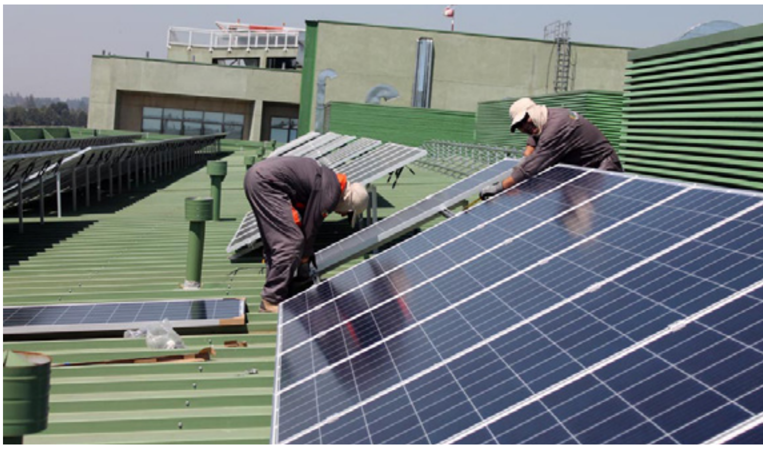

A public hospital in Chile generates electricity with solar panels. Photo: Complejo Asistencial "Víctor Ríos Ruiz".

Table 2 – Scopes

Scope 1: Direct GHG emissions	Scope 2: Electricity/steam/heating/cooling indirect GHG emissions	Scope 3: Other indirect GHG emissions
Direct GHG emissions occur from sources that are owned or controlled by the institution, such as emissions from combustion in owned or controlled boilers, furnaces, or vehicles; emissions from on-site waste treatments; or fugitive emissions of anesthetic gases or cooling equipment.	Scope 2 accounts for GHG emissions from the generation of the electricity/steam/heating/cooling consumed by the institution (purchased or otherwise brought into the organizational boundary of the institution). Scope 2 emissions physically occur at the facility where these items are generated.	Scope 3 is a reporting category that allows for the treatment of all other indirect emissions. Scope 3 emissions are a consequence of the activities of the institution but occur from sources not owned or controlled by it. Some examples of scope 3 activities are business travels, patient and employee commuting, electricity transmission and distribution losses.

Source: Based on GHG protocol definitions[5].

[ii] GHG Protocol - A Corporate Accounting and Reporting Standard[5]; Global Protocol for Community-Scale Greenhouse Gas Emission Inventories[9].

Figure 1 – Scopes

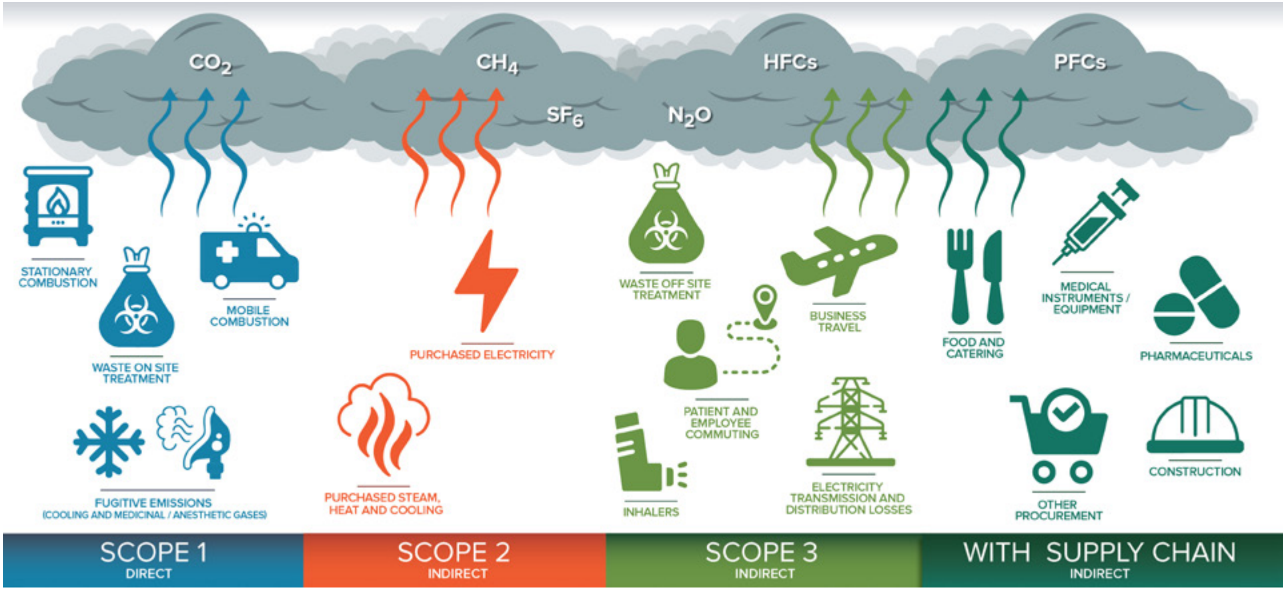

Source: Developed by Health Care Without Harm for this document.

Figure 2 – Global health care footprint split by scopes

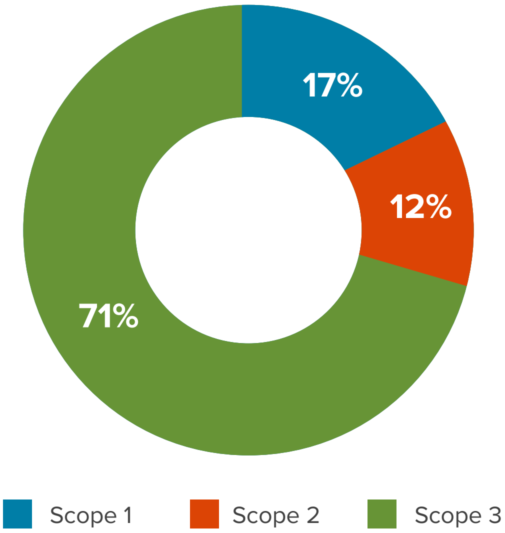

Source: Health care's climate footprint, Health Care Without Harm, 2019[3].

How to estimate GHG emissions

There are two framework approaches to calculate emissions in a GHG emissions inventory: the top-down and the bottom-up.

The top-down is commonly used for national, territorial or sectoral GHG inventories, as it considers national data from official statistics and/or environmentally extended input–output databases. One advantage of a top-down method is that it provides a zero-leakage order-of-magnitude assessment of the emissions inventory of large organisations or areas of economic activity, such as national health systems.

The bottom-up is commonly used for organizational or project GHG inventories, as it considers local or facility-level specific data. Advantages of a bottom-up method include the provision of highly granular information regarding the footprint of an activity. Climate Impact Checkup tool uses the bottom-up approach.

For more information on these approaches, see the Health Care Without Harm & ARUP publication: Designing a Net Zero Roadmap for Healthcare: Technical Methodology and Guidance[7].

Methodologies for estimating GHG emissions are based on the 2006 IPCC guidelines for national greenhouse gas inventories[8] and the corporate accounting and reporting standards of the GHG protocol[5].

The general equation of these methodologies can be expressed as:

GHG emissions=activity data x emission factor

Activity data is a quantitative measure of an activity that results in GHG emissions that takes place during a given period of time (such as volume of gas used, kilometers driven, or tons of waste sent to the landfill). An emission factor is a measure of the mass of GHG emissions relative to a unit of activity. For example, estimating CO_2 emissions from the use of electricity involves multiplying data on kilowatt hours (kWh) of electricity used by the emission factor (kg CO_2/kWh) for electricity, which will depend on the technology and type of fuel used to generate the electricity[9].

Commonly activity data are specific values for each institution, as opposed to emission factors, which are usually national values published by reference bodies or default values from the bibliography.

Principles of GHG accounting

Based on the GHG protocol[5], the following principles are considered good practice when estimating and reporting the emissions of an institution:

Table 3 – Principles of GHG accounting

**Relevance**	Ensure GHG inventory appropriately reflects GHG emissions of the institution and serves the decision-making needs of users – both internal and external.
**Completeness**	Account for and report on all GHG emission sources and activities within the chosen inventory boundary. Disclose and justify any specific exclusions.
**Consistency**	Use consistent methodologies to allow meaningful comparisons of emissions over time. Transparently document any changes to the data, inventory boundary, methods, or any other relevant factors.
**Transparency**	Address all relevant issues in a factual and coherent manner, based on a clear audit trail when applicable. Disclose any relevant assumptions and make appropriate references to the accounting and calculation methodologies and data sources used.
**Accuracy**	Ensure that the quantification of GHG emissions is systematically neither over nor under actual emissions, as far as can be judged, and that uncertainties are reduced as far as practicable. Achieve sufficient accuracy to enable users to make decisions with reasonable assurance as to the integrity of the reported information.

Language differences

Common items often have different names in different countries. In this tool the names used for fuels follow IPCC nomenclatures.

Table 4 – Names of fuels

Climate Impact Checkup	Other names
Natural gas	Compressed natural gas (commonly used in vehicles)
Liquefied petroleum gases	-
Gasoline	Petrol, naphtha, motorgasoline
Gas/diesel oil	-
Fuel oil/bunker	-
Kerosene	-
Coal	-
Wood	Wood waste, solid biomass
Biodiesel	-
Biogasoline/bioethanol	Ethanol

Waste streams considered in the tool, are based on a combination of names from IPCC and the Hippocrates[iii] waste tool, as shown in the following table.

Table 5 – Names of waste streams

Climate Impact Checkup	IPCC guidelines	Hippocrates waste tool
Non-hazardous/general health care waste	Municipal solid waste	Non-hazardous or general health care waste
Clinical mix (infectious/ biohazardous and hazardous)	Clinical mix	Infectious/biohazardous waste and hazardous chemical waste
Hazardous	Hazardous	Hazardous chemical waste

Measurement units in the tool

Data requested

The most commonly used unit may differ among countries, so values can be entered in different units in most categories. When more than one unit is available, it is important to select from the scroll-down menu the unit according to the value.

Some units used in the tool include:

- Volume: liters, gallons, m^3
- Mass (also called weight): kilograms (kg), pound (lb), metric ton
- Natural gas: m^3, mmBTU, SCF, Therm (USA)[iv]

According to the unit selected, the tool will automatically make the conversions needed. The equivalences between units are available in the "lists" section.

For distance values (used in scope 3 transport categories), data should be entered in kilometers.

[iii] Hippocrates is the online database and reporting platform for members of the Global Green and Healthy Hospitals Network.

[iv] This unit corresponds to the Therm, mostly used in the United States, not to the Thermie.

Emissions

Emissions are expressed in:

- Kg of the greenhouse gas (for instance, kg of CH_4)
- Kg of CO_2e
- Tons of CO_2e: in the results section

When expressed in terms of CO_2e, the GWP values used for the conversions are listed in Annex I.

Biogenic emissions

CO_2 emissions from biofuels such as biodiesel, bioethanol, and firewood are considered neutral in net terms (the amount of CO_2 the biomass captured throughout its life is equal to the amount it emits when burned). These are not accounted for but expressed as zero.

If your country has mandatory biofuel blending in the commercial fuels, the tool will automatically divide the amount of fuel entered. For instance, consider a 15% blend of bioethanol in gasoline. If you consume 10 liters of gasoline, 8.5 liters will be treated as this fossil fuel and 1.5 liters as bioethanol.

Institutional arrangements

Estimating a health care institution's carbon footprint requires information from many different sources. For this reason, time and effort at the beginning of the process should focus on establishing the institutional arrangements for data collection and action planning.

Institutional arrangements will facilitate the collection and generation of information needed to make calculations and ensure these actions continue to evolve in time. When establishing institutional arrangements, consider the following activities:

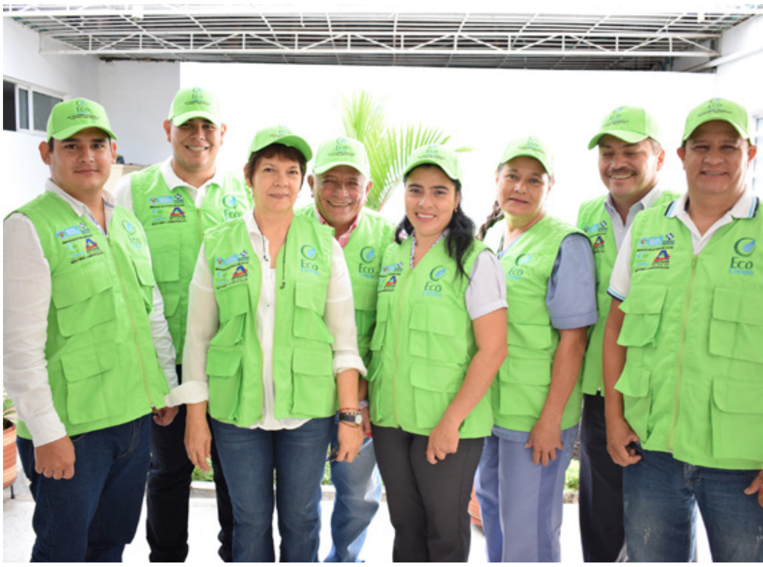

Environmental management leaders at E.S.E Centro in Cali, Colombia. **Photo:** E.S.E Centro

- Communicate the initiative to the hospital's main authorities and seek their support.
- Identify the people responsible for necessary data. Invite them to a meeting to introduce the project and request data and collaboration.
- Consider communicating possible outcomes in indicators or units of measurement that are attractive to a variety of stakeholders, such as savings, safety, and workforce engagement.
- Communicate the project to the entire institution (to obtain staff involvement it is necessary for everyone to know what is happening). This can be done through posters, institutional newsletters, or general meetings.

What is in the tool

(and what may be included in future versions)

The tool developed by Health Care Without Harm Global currently covers the following categories of GHG emissions sources:

Table 6 – Health care facilities carbon footprint. Health Care Without Harm tool

Scope 1	Scope 2	Scope 3
• Stationary combustion<br>• Mobile combustion<br>• Fugitive emissions (cooling and medicinal/anesthetic gases)<br>• Waste on site treatment	• Purchased electricity<br>• Purchased steam, heat or cooling	• Extra supply chain<br>• Business travel<br>• Electricity transmission and distribution losses<br>• Inhalers<br>• Waste off site treatment<br>• Employee commuting<br>• Patient commuting

These sources were selected because of their relevance to general emissions sources and health care sector (such as fugitive emissions from medicinal gases) as well as the ability to get representative and accurate data.

As the tool is used over time, further upgrades are expected. The feedback Health Care Without Harm receives from its users is essential. Errors may be identified and need correction, and other features may also need to be improved.

In the future, more sources of emissions will be added along with local/specific data. Some sources currently being analyzed include wastewater treatment and electromobility.

However, from an institution perspective, it is difficult to specifically determine some sources' GHG impact when they are deeply connected to local parameters of production and interconnected with other activities.

How to use the tool

Climate Impact Checkup's carbon footprinting tool was developed to calculate GHG emissions of health care institutions in any country in the world. This is done using facility-level data relating mainly to energy consumption, transport, and waste management, as well as other gases relevant to the sector, such as anesthetic gases, cooling gases, and emissions produced by inhalers.

User profile

Users of the tool may include:

- health care facilities of any level of complexity and in any location,
- state health care systems,
- national health care systems, and
- research institutions, such as universities.

Health care facilities that are members of the Global Green and Healthy Hospitals network have access to a more complete version of the tool. Members can see graphics, access a benchmarking report, and print the results section. Non-members will have a limited access to the tool.

How to access the tool

The tool is available for GGHH members in three different ways:

- Online/web based
- Offline/Excel based

Online and offline versions of the tool

This guideline was developed based mainly on the online version of the tool, which can be viewed in Annex II. However, all sources described in this document have their analog section in the offline version. The main differences in the offline Excel version are:

- Profile information is requested in sheets for **step 1** and **step 2**
- Some sources have auxiliary forms, such as cooling gases, medicinal/ anesthetic gases, business trips, employee commuting, and patient commuting.

Definition of institutional boundaries for emissions estimation

If an institution consists of more than one health care center, it will have to define whether the carbon footprint will be estimated for all units together, only for some, or for each facility individually. In any case, it is important for the scope to be clearly defined and stated.

Important: This tool collects information at facility level. GGHH members can create a System profile and an account per facility and report per facility for one system.

Profile information

When using the tool, start by completing profile information. Many institutions will have already completed this section, while for others this will be their first time entering data. No matter the case for your institution, some data should be updated annually.

This section requests general data on the institution and its context, such as name, city, years in service, sub-national region (only for countries where the electricity grid varies between regions), hours of operation, type of institution, and weather conditions (temperature and humidity). This information not only provides a description of the institution but also helps with estimations (for example, temperature affects the decomposition of waste in final disposal sites).

In addition to this, some data depends on the year and should be completed annually:

- Occupied beds (annual average)
- Full-time equivalent employees (FTE)
- Patients (annual total)
- Building covered area (in m^2)

Occupied beds (annual average): This refers to the number of inpatient beds the hospital has. However, if the hospital normally operates under capacity, indicate the average quantity that is occupied. For example, if a facility with 500 available beds in 2019 had an 85% annual occupation rate, it will have 425 occupied beds in 2019.

Full-time equivalent employees (FTE): This indicator allows facilities to sum up and compare part-time and full-time employees by converting the hours worked by several part-time employees into hours worked by full-time employees.

Patients: This parameter includes all patients (both inpatient and outpatient) who come to the institution during each year. This includes medical consultations, laboratory and imaging studies, surgical interventions, and other reasons.

Using these parameters, it is possible to build indicators such as emissions per patient or emissions per bed and allow users to make comparisons in similar institutions.

Introduction and general data

Most institutional data used in the tool is taken from the facility's profile in Hippocrates. However, additional information used for estimating GHG emissions should be entered in this section.

Data requested:

- Year: It is necessary to select a year. This cannot be the current year; it should be the year for which data is complete. Estimates are done on an annual basis, so data for a complete year is required.

> If your country's reporting is generally made under a financing year scheme, then select the first year of this period as the year for the carbon footprint. For instance, if you are reporting for the 2017/2018 financial year, you should select 2017 in the tool.

Notation keys

Very important: In these sections the information needed to estimate emissions is requested. However, the institution might not complete all sources for various reasons. The first step in each category is to select the treatment each source will receive using the following notation keys:

Table 7 – Notation keys

Notation key	Description
**Estimated**	Emissions in this category are estimated
**Not estimated / Data not available**	Emissions occur but were not estimated due to lack of data
**Not estimated / Complex**	Emissions occur but were not estimated because data management is very complex
**Not occurring**	The activity or process does not exist within the institution

The use of notation keys will help institutions and Health Care Without Harm to understand data gaps, training needs, or technical assistance to track some sources of emissions.

If you select “not estimated” or “not occurring,” we encourage you to enter additional information in the text box available to add comments. For instance, if your institution has a 100% renewable electricity purchase contract, you will select “not occurring” in the category “purchase of electricity” and please clarify its reason in the text box.

Testing or reporting with Checkup?

When starting a new form, users need to specify why they are completing the tool by selecting one of the following options:

- Calculating and reporting emissions to Health Care Without Harm
- Learning how to use the tool
- Creating test scenarios

In the last two options, users can fill out the form with hypothetical information to better understand how the tool works or compare emissions between different intervention alternatives. For example, if a hospital has decided to buy a new air conditioner, it can compare the emissions caused by the cooling gases from each option under consideration using the tool. By specifying the purpose of the submission, the system separates real reports from test data.

Scope 1

Stationary combustion

This section estimates GHG emissions resulting from the burning of fuels in stationary processes. Stationary combustion takes place in devices that do not move, such as boilers, hot water tanks, stoves, water heaters, backup generators, on-site waste incinerators, and autoclaves.

Data needed

- Total amount of each type of fuel consumed for the year
- Unit of the value, which should be selected from the options provided in the scroll-down menu.

Where can the user find these data?

Natural gas bills, quantity of LPG tanks bought during the year, fuel purchase receipts.

Mobile combustion

Mobile combustion refers to GHG emissions resulting from burning fuel in vehicles belonging to the health center. These vehicles can be ambulances, cars, or motorcycles. Emissions resulting from employee commuting and business trips are accounted for under scope 3.

Natural gas in kWh

Some institutions measure the gas they purchase in kWh. In these cases, the unit selected should be m^3 and the value converted by dividing it by 9.33[v].

Example:

Consumption of natural gas (in kWh)= 300

$$\text{Consumption of natural gas (in } m^3\text{)}= \frac{300}{9.33} = 32.15$$

So, 32.15 will be the value entered for natural gas consumed and m^3 the unit chosen.

Data needed

- The amount of gasoline, diesel, and compressed natural gas consumed in the institution during the year.
- For every value entered, the unit should be selected from the options provided in the scroll-down menu.

[v] This conversion considers 3.6MJ = 1kWh, the density of natural gas is 0,7 kg/m^3 and its low heating value, 48 MJ/kg. By using this conversion factors, it results that:

$$1m^3 \text{ natural gas} = \frac{1m^3 \times 0.7 \frac{kg}{m^3} \times 48 \frac{MJ}{kg}}{3.6 \frac{MJ}{kWh}} = 9.33 \text{ kWh}$$

The total amount of consumed/purchased fuels should be entered. If your country uses a mandatory biofuel blend, the tool will automatically divide the amount of fuel entered. For instance, consider a 15% blend of bioethanol in gasoline. If you consume 10 liters of gasoline, 8.5 liters will be treated as this fossil fuel and 1.5 liters as bioethanol.

Where can the user find these data?

Fuel purchase receipts, bills, fleet vehicle logs.

Fugitive emissions

Cooling & fire suppression

This category estimates unintentional emissions of gases used for cooling and insulation, as well as those used in fire extinguishers. Emissions are estimated based on the quantity of gas reloaded annually in equipment such as air conditioners and fridges.

Data needed

In each row the following data should be completed:

- **Type of equipment:** Select an option from the scroll-down menu.
- **Type of gas used:** Select an option from the scroll-down menu.
- **Quantity reloaded:** Enter the value in numbers.
- **Unit:** Select an option from the scroll-down menu.

If the quantity of rows is not enough for your entries, press the "Add another row" button to add as many as you need.

Where can the user find these data?

Receipts of equipment bought and gas recharges, environmental management audit reports (as the use and consumption of these gases are also frequently monitored by this area), requests to the maintenance department.

Equipment retired

In addition to the equipment recharged in the year chosen, equipment retired in that period should also be taken into account. The first thing to check is whether the amount of gas it contains was already accounted for in any previous carbon footprint. Second, you will need to figure out the density of the cooling gas it contains. With this information, the options to follow are:

- If the amount of gas was already included in a previous carbon footprint and not recycled, do not enter this quantity again or it will be double counted.
- If the amount of gas was already included in a previous carbon footprint and recycled or recharged in new equipment, this should not be accounted for in the emissions of that year or it will be double counted.
- If the amount of gas was not included in a previous carbon footprint, the quantity of gas that the equipment contains should be included as a recharge that year so emissions will be reported. It does not matter if the gas is recycled in other equipment or sent to final disposal, as in both cases it will end up released into the atmosphere.

Medicinal / anesthetic gases

Some GHGs (such as N_2O) are used for medicinal and anesthetic purposes. These activities' emissions are accounted for in this category.

Data needed

- Total quantity delivered or the number and size (in ml) of the bottles and cylinders used for each gas. The data should be the amount delivered, not purchased. We recommend to report total amount delivered plus total amount discarded as waste due to expiration date or a different reason for discarding as waste without use.

Where can the user find these data?

Operating room services, surgical services.

Anesthetics do not undergo changes in composition in the human body. This means the amount ingested is equal to that which is later eliminated. Therefore, to calculate emissions in this category, the annual amount of each gas consumed is used directly.

Scope 2

Purchase of electricity

This section estimates GHG emissions that result from the burning of fuels in thermal power plants to generate electricity consumed in the institution.

Data needed

- Total of electricity consumed (in kWh) in your institution for the chosen year.

Where can the user find these data?

Utility bills or electricity meters.

Grid emission factors or GHG emission intensity of power generation

When estimating projected GHG reduction from a mitigation project, the grid emission factor of a country is calculated using UNFCCC's tool to calculate emission factors for an electricity system[10].

Grid emission factors can be classified as two types: project factors (also called construction, operating margin, and combined) and average factor (or carbon intensity).

The factors help determine what a certain action (typically energy efficiency measures or renewable energies) saves thanks to its implementation. The basis of this estimate is trying to predict what type of electricity generation this project will replace. In other words, what type of energy generation will be avoided thanks to this project.

On the other hand, when consuming electricity from the grid, emissions depend on the average composition of power generation. This is the emission factor used in the tool and can be called the "average emission factor" or "GHG/carbon intensity of power generation."

Purchase of steam, heat, and cooling

District heating or cooling networks are a common public service in some countries people and institutions buy steam, heat, or cooling in a similar way to grid electricity worldwide. This section accounts for GHG emissions that may be released if fuels are burnt or electricity consumed to produce steam, heat, or cooling purchased, depending on the source.

Data needed

This section is divided into two groups of countries in the tool. On the one hand, Canada and the US, where there are systematized emission factors for this category. On the other hand, all the other countries, where there is no reference information to be used.

Canada and the United States:

- Quantity consumed of each item (in kbtu):
  - Steam
  - Hot water
  - Chilled water: electric driven chiller
  - Chilled water: absorption chiller using natural gas
  - Chilled water-engine-driven chiller using natural gas

Where can the user find these data?

- Bills or meters.

Other countries:

- Emissions (in $kgCO_2e$): There is not systematized data for other countries, so these emissions per item should be directly entered in $kgCO_2e$.
- Source: Geothermal, grid electricity, solar thermal, etc.
- Description of other information about these emissions including emission factor value, reference, etc.

Where can the user find these data?

Ask the maintenance or environmental department of the institution if they already estimate these emissions. If they do not, other possible sources for this information are the provider company of the steam or cooling, the environmental local authorities, tools available on the internet, etc.

Scope 3

Transport-related categories

This refers to emissions resulting from the burning of fuel for the transport of people related to the institution. There are three categories:

- Business trips for staff (conferences, workshops, seminars, meetings in places outside of the institution)
- Employee commuting
- Patient commuting

Data needed

Even though there are three different categories for emissions, information requested in the tool is similar for each case.

For every business trip or commute (employees or patients), a single row should be completed with the following data:

- **Transport:** Select an option from the scroll-down menu.
- **Distance:** Enter the number value in kilometers. You only need to enter the distance one way; the tool will multiply by two to consider the round trip.
- **From/to:** This refers to the starting point and final destination of the trip ***(Consider only business trips).***
- **Weekly frequency/number of days a week:** How many times a week does this commute occur? ***(Consider only employee commuting).***
- **Reason:** Select an option from the scroll-down menu ***(Consider only patient commuting)***.

In the business trip category, distances might be longer and may vary depending on the type of transport (plane or bus/car), so an online distance calculator can be used. For instance, this distance calculator.

Because it can be difficult to acquire data for patient commutes, a sampling approach is proposed. Before completing the data, the duration of the sample must be selected from the scroll-down menu on the main page. Options include a week (7 days), a month, or a year. Each institution should design its own sampling process.

Inhalers

This section estimates GHG emissions that result from the use of metered dose inhalers (MDI) and dry powder inhalers (DPI).

Data needed

As data may come from different areas, emissions in this category are classified under three subcategories of inhaler acquisition: dispensed at the institution, sold/delivered in the central pharmacy (for hospitals with a pharmacy inside the facility), and prescribed by doctors.

For each of these subcategories, it is necessary to enter the number of inhalers and its quantity of doses and select its type (MDI or DPI).

For prescribed inhalers, it is also requested to list the percentage of inhalers that are bought and not included in other subcategories. There is a default value suggested but it can be modified if it does not reflect the situation of the institution.

Sistematization

Individually collecting and systematizing this information can be difficult. One way to make this task easier is by using an online service to create and share surveys (such as Google Doc forms). Create a question for each type of data requested, remembering to add a scroll-down menu with the same options offered by the tool when applicable. Distribute the survey by sharing the link with all members of the hospital or patients, explaining the reason behind the request so they feel encouraged to complete it and setting a deadline for submissions.

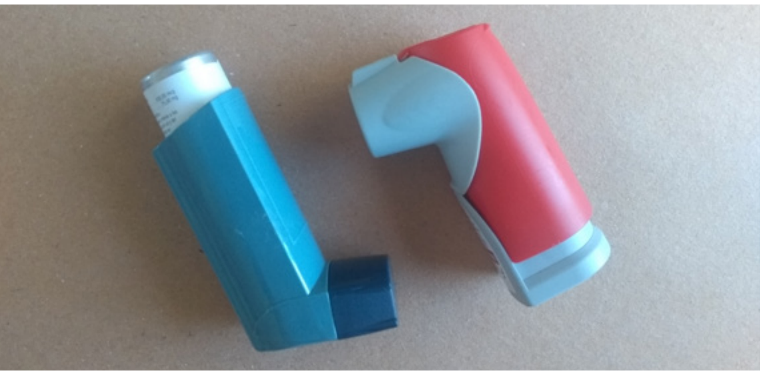

Inhalers at Hospital Universitario Austral, Argentina.
Photo: Dr. Verónica Torres Cerino.

Where can the user find these data?

Medical or prescription records, central pharmacy logs.

Electricity transmission and distribution losses

This section estimates GHG emissions that result from losses in the transmission and distribution of electricity consumed by the institution. When entering the data requested in the “purchase of electricity” category in scope 2, these emissions are automatically estimated; no extra data is needed.

Extra supply chain

The supply chain consists of all kinds of goods and services a hospital or a health care facility purchases from third parties (providers or suppliers) and this is one of the reasons why this category of emissions is classified in the scope 3.

The supply chain is one of the main components of scope 3 emissions in the health care sector. Moreover, the Global Road Map for Health Care Decarbonization[11] (which provides pathways to achieve a climate resilient health care sector with zero emissions), defined the supply chain decarbonization as one of the main pathways towards these objectives, as well as a key factor for pandemic preparedness. See more about the Roadmap in Part 2 of these Guidelines.

By calculating their supply chain emissions, health care facilities and systems will be able to make further steps towards a more sustainable, low-carbon, and resilient health sector.

Figure 3: Health care's global emissions by supply chain categories.

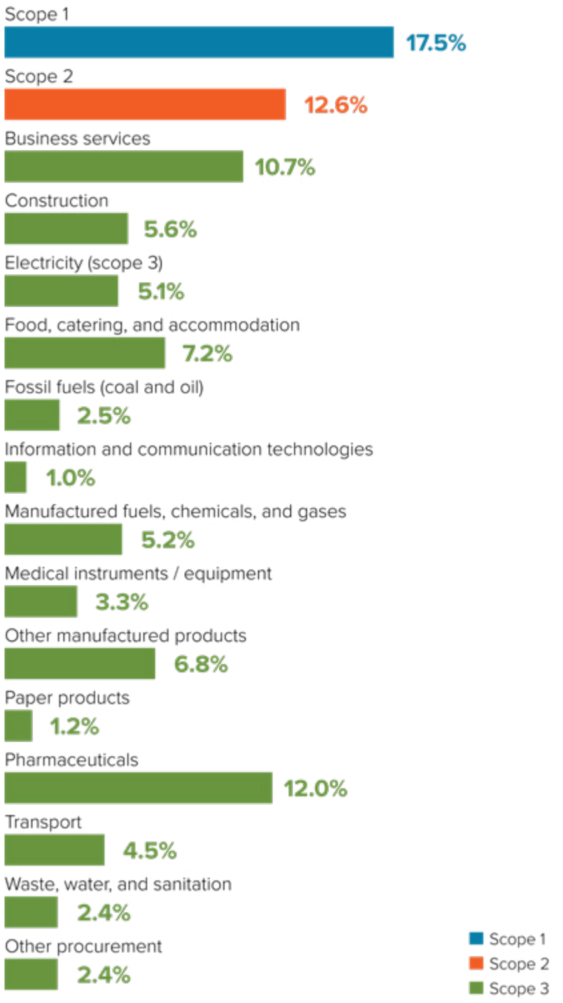

Source: HCWH and ARUP. Global Road Map for Health Care Decarbonization[11] (2020)

How does the extra supply chain section of Checkup calculate the GHG emissions?

Information of the World Input and Output Data Base (WIOD)[vi] and its environmental accounts[vii], is grouped in spend categories (Figure 3). The categories not covered in previous sections are estimated by the extra supply chain (Annex III). The tool takes the annual spending provided by the user for each extra supply chain category with their corresponding emission factor. These emission factors are established in carbon intensity (kgCO_2e/USD) and they are taken from the WIOD.

Data needed

- Local currency: select an option from the scroll-down menu.
- Annual average US dollars conversion rate for the selected year.
- Annual spending for each supply chain category in Table 9 (Annex III). Values must be entered in the local currency.

Where can the user find these data?

- The US dollar conversion rate can be obtained preferably from an official local authority or from an official US authority like the Internal Revenue Service (IRS) which publishes annual conversion rates per currency/country.
- The annual spending can be found in suppliers' receipts or invoices, in the procurement department or audits reports and/or in the procurement software.
- The health description and usage columns in Table 9 (Annex III) provide a description of each category. This helps the user to know how to classify each expenditure from each supply chain category. For further information, consult the Global Road Map for Health Care Decarbonization.[11]

Features of the extra supply chain section of Climate Impact Checkup tool

- It is integrated in the tool to include supply chain in the whole GHG inventory calculation and results.
- It is a spend-based tool; therefore, it does not require purchased quantities per unit of product/service, but the yearly spending for each category.
- It includes 11 supply chain categories (Figure 3) not covered in previous sections, with emission factors for 43 countries and average factors for the rest of the world.
- The categories that are not included in this section of the tool because they are covered in previous sections, are: electricity, fossil fuels (coal and oil), waste products and recycling, and transport.

The supply chain calculation results

- Categories are ranked by their GHG emissions share to identify easily the hotspots in the supply chain.
- Results include a chart with the top five supply chain categories based on their emissions (hotspots).
- The tool provides tables and graphics in which the supply chain results are compared with the whole GHG inventory.
- Results also include the share of the spending and the share of each category in the whole supply chain calculation.

Completeness cross-check

Because some supply chain categories are covered in previous sections and not included the Extra supply chain section, a completeness cross-check is proposed to ensure that all emissions are accounted for. To conduct it, these instructions must be followed right after having completed the data needed described above:

[vi] WIOD: Project funded by the European Commission and with participation of 11 European organisations among universities and research or studies centres. See more at: https://www.rug.nl/ggdc/valuechain/wiod/

[vii] https://publications.jrc.ec.europa.eu/repository/handle/JRC116234

1 - Fill in the institution expenses for each category shown in Table 8, which includes the categories not reported under the extra supply chain.

2 - Fill in the total spend of your institution without the staff salaries.

3 - Note the difference between the result of the sum of the spends of the supply chain categories (extra supply chain plus the ones covered in the completeness cross-check), and the total spend of your institution. If the difference is considerable, you should revise the spend data entered per category.

As the Climate Impact Checkup tool is always under a continuous improvement process, the following two multiple choice questions are asked with the aim of gathering information about the cross-check section:

- Did you complete the completeness cross check?
- Did you find it useful?

Table 8 – Grouped categories, WIOD definition, and WIOD subcategories for the completeness cross-check.

Grouped sectors (Category)	WIOD definition	WIOD subcategories (Health usage)
**Electricity**	Emissions associated with the transmission and distribution of electricity purchased by the health sector, as well as the electricity generated within the sector's own supply chain	Electricity, gas, steam and air conditioning supply
**Fossil fuels (coal and oil)**	Emissions associated with the production of fossil fuel products procured by the health sector for uses including boilers, generators, and vehicles. These emissions are those generated in the production of these fuels.	Manufacture of coke and refined petroleum products
**Waste products and recycling**	Emissions associated with treatment and waste disposal and recycling	Sewerage, Waste collection, Treatment and disposal activities, Materials recovery, Remediation activities and other waste management services.
**Transport**	Emissions from transport services purchased by the health sector, covering freight and passenger transport	Land transport, Transport via pipelines, Water transport and Air transport

Source: HCWH and ARUP. Global Road Map for Health Care Decarbonization (2020)[11]
Annex A: Technical Report, Table A.4 and Table A.5. 2021.

Waste

This section estimates emissions of management, treatment, and disposal of waste produced in the institution, regardless of whether these activities take place inside or outside the facility.

Composition of non-hazardous, general health care waste produced by the institution is a parameter used to estimate the emissions of some categories. If this information is available for your facility, answer "yes" when asked if you know the composition of your non-hazardous/general health care waste, and enter the value of each material (in percentage). Otherwise, say "no," and default values for your country will be used.

There are three categories under waste: solid waste disposal, composting, and incineration.

When these activities take place in the institution (*in situ*), emissions are accounted for in scope 1. In contrast, when emissions occur outside the institution (*ex situ*), they are accounted for in scope 3. Before entering data in each category, select the appropriate option.

Where can the user find these data?

Environmental management department, maintenance staff.

> If you find significant differences with estimations made with national data from your country, please let us know as we will continue working to improve this section in the future.

Solid waste disposal

This section estimates methane (CH_4) emissions occurring due to anaerobic processes that take place in solid waste disposal sites (landfills or garbage dumps).

The emissions of this category depend on the combination of several parameters such as the composition of the waste, the characteristics of the disposal site and the weather conditions of the place over the time. The ultimate goal is to forecast the quantity of methane released to the atmosphere due to the waste buried. For this reason, this estimation may be different from others made with national emission factors, when available.

Data needed

- You will need the amount of waste that is sent annually to final disposal at your institution, including those that were autoclaved. (The composition of autoclaved waste likely differs from non-hazardous or general health care waste, and it will not result in significant impact. This can be considered a conservative approach, as it is generally composed of materials that do not generate CH_4 emissions under anaerobic processes.)
- Select the unit of the value from the options provided in the scroll-down menu.
- Type of waste disposal site (landfill or garbage dump). If this information is not available, select "I do not know."

> All the calculation methods, equations and default data used in this category are available in the 2006 IPCC guidelines[8], Volume 5 Chapter 3.

Waste disposal GHG emissions are affected by temperature. Remember to report accurate data in the Profile.

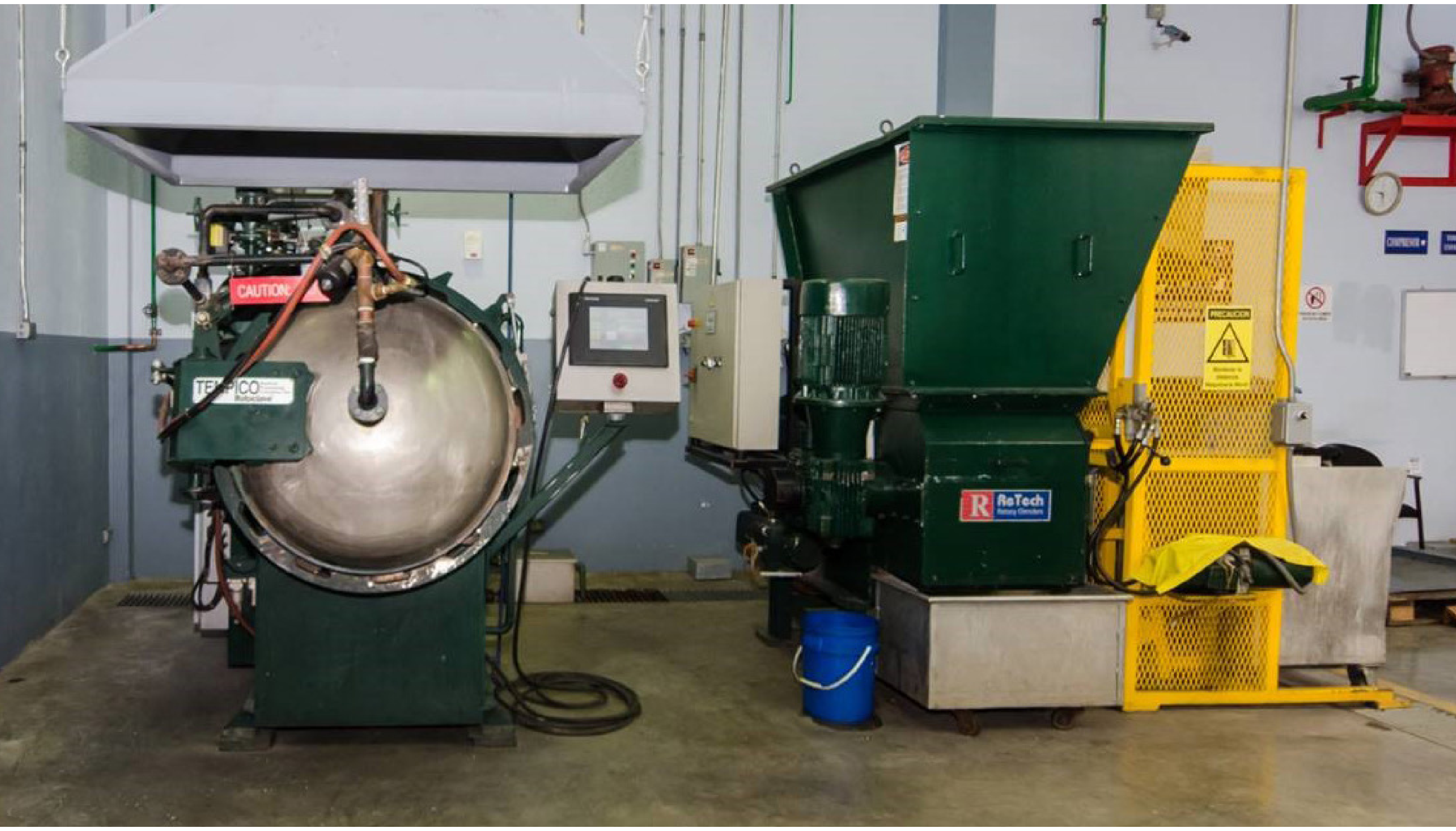

Autoclave at Clinica Biblica, Costa Rica. Autoclaving emits and pollutes less than waste incineration. Photo: Clínica Bíblica.

Where can the user find these data?

Administration department, environmental management department, maintenance staff, waste treatment company records, logs, and receipts, waste manifests.

Composting

Methane (CH_4) and nitrous oxide (N_2O) emissions are released during solid waste composting (biological treatment) processes. Carbon dioxide (CO_2) emissions also take place, but because it is the organic fraction of the waste, these emissions are considered biogenic (neutral in net terms) and therefore not calculated. See biogenic emissions box, page 13, for more information.

Data needed

- Amount of waste that is annually composted (or composted off site) at the institution.
- Select the unit of the value from the options provided in the scroll-down menu.

Where can the user find these data?

Administration department, environmental management department, maintenance staff, waste treatment company records, logs, and receipts, waste manifests.

Incineration

This section estimates carbon dioxide (CO_2) emissions that result from combustion of the fossil carbon fraction of wastes. CO_2 emissions from the organic carbon fraction are considered biogenic (neutral in net terms) and therefore not calculated. See biogenic emissions box, page 13, for more information.

Data needed

- Amount of each type of waste (non-hazardous/ general health care waste, clinical mix of biohazardous and hazardous, and hazardous) that are annually incinerated (on site or off site incineration) at the institution.
- For each category of waste, select the corresponding unit from the options provided in the scroll-down menu.

Where can the user find these data?

Administration department, environmental management department, maintenance staff, waste treatment company records, logs, and receipts, waste manifests.

Emission factors and other parameters: Where do they come from?

Many emissions factors are needed to calculate GHG emissions, along with activity data provided by users.

This technical data comes from sources described in the references sheet, which can be downloaded from the intro page in the online version of the tool and in the References sheet in the offline version of it.

Quality checks

When doing analysis that requires many types of data from different sources, it is common to make mistakes. Therefore, these checks are fundamental to identify, avoid, and prevent errors in order to ensure the quality of the estimations, as well as improve them in the future. This a non-exhaustive list of activities that can be carried out based on the GHG protocol[5]:

- Check the input data for transcription errors
- Check if units are properly labeled and correctly carried through the calculations
- Check whether input data units are selected correctly and appropriately labeled
- Check consistency of time series inputs and calculations

- Check that changes in data or methodology to obtain it are documented
- Confirm that bibliographical data references are registered
- Check that assumptions and criteria for selection of boundaries, base years, activity data, and other parameters are documented.

Good practices: Data sources

Completing the "data sources" sheet available in the Excel version of the tool is highly recommended, even though the information it requests is not directly used by the tool to estimate emissions.

At first it may appear to be a labor-intensive activity without any concrete results, but having metadata (unit, year, reference, category) for each value used to estimate carbon footprint will be helpful when repeating this activity in subsequent years and verifying transparency and quality of calculations. See the **principles of GHG accounting** section for more information.

Results and comparing data

This section presents results along with different ways to better understand them:

- Numeric values in terms of tons of CO_2e per source, scope, and total
- Pie charts, bar graphs, and other types of graphics
- Indicators (emissions per occupied bed, patient and employee). **These are estimates combining emission results with parameters of the institution. Because of this, it is important to complete the data requested in the profile section. Otherwise, these indicators cannot be estimated**.

There are differences in how GHG emissions are distributed in various health care facilities. For example, in Figure 4, differences between levels of complexity in the data collected in the LAC region by Health Care Without Harm in 2019 are apparent (2018 data). The location, climate, type of facility, number of patients received by year, percentage of occupied beds, electricity production, and other variables affect the size and sources of total GHG emissions produced by a health care facility. The tool requests general data to gain a better sense of the facility's context and includes normalizers to compare data from different facilities.

Figure 4 – GHG emissions per scope in different levels of complexity. LAC GGHH member data

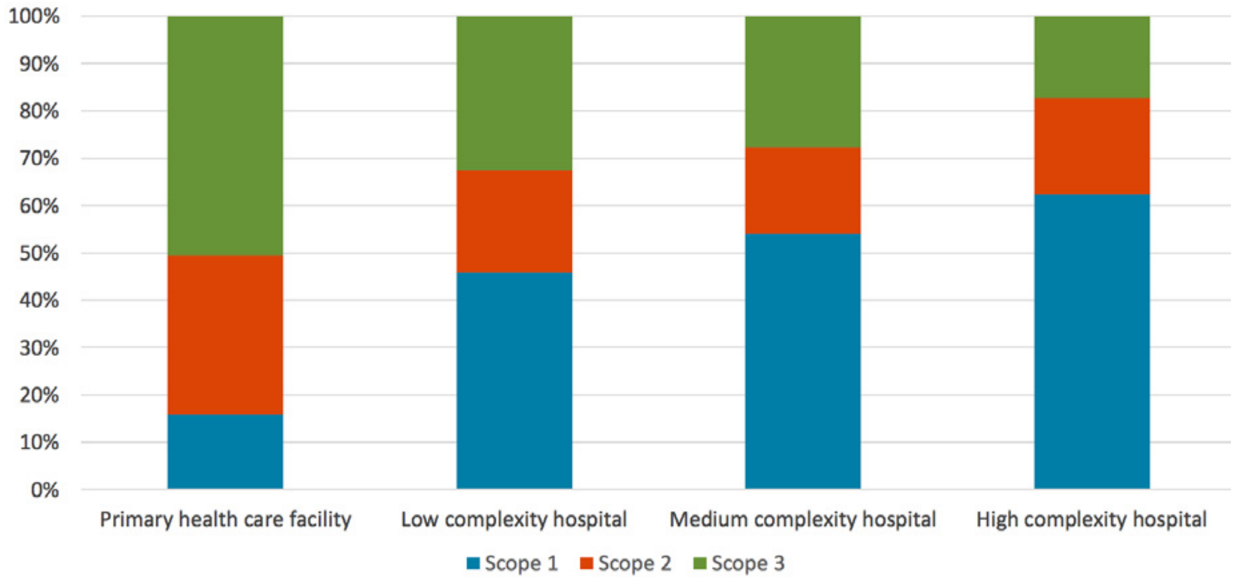

Glossary[viii]

Carbon neutrality and net zero: Net zero and carbon neutrality are terms used to mark the point where an entity has achieved a balance between their emission reduction efforts and the compensation of remaining or residual emissions by engaging in emission removal activities (e.g., reforestation efforts or carbon capture) and/or purchasing an equivalent amount of offsets. Many offset schemes are highly questionable in their efficacy to achieve absolute emissions reduction, while also raising a series of ethical questions. Still, the term net zero is often preferred to carbon neutrality because it is more stringent and covers a wider scope of GHG emissions. It points to a faster pace of decarbonization across all scopes and then only considers compensation mechanisms for emissions that are particularly difficult to mitigate despite all the targeted interventions, investment, and focus.

Climate change: This refers to a change in the climate that can be identified by changes in the mean or variability of its properties and that persists for an extended period, typically decades or longer. Climate change may be due to natural internal processes or external events, such as modulations of the solar cycles, volcanic eruptions, and persistent anthropogenic changes in the composition of the atmosphere or land use. UNFCCC defines climate change as “a change of climate which is attributed directly or indirectly to human activity that alters the composition of the global atmosphere and which is in addition to natural climate variability observed over comparable time periods”. UNFCCC thus makes a distinction between climate change attributable to human activities and climate variability attributable to natural causes.

Direct GHG emissions: These are emissions from sources owned or controlled by the institution.

Global mean surface temperature: This refers to an estimate of the global mean surface air temperature. For changes over time, only anomalies as departures from climatology are used, commonly based on the area-weighted global average of the sea surface temperature and land surface air temperature.

Global warming: Global warming refers to the gradual increase, observed or projected, in global surface temperature, as one of the consequences of radiative forcing caused by anthropogenic emissions.

Global warming potential (GWP): This index represents the combined effect of the differing times greenhouse gases remain in the atmosphere and their relative effectiveness in absorbing outgoing infrared radiation.

Greenhouse effect: This is the radiative effect of all infrared-absorbing constituents in the atmosphere. GHGs, clouds, and (to a small extent) aerosols absorb terrestrial radiation emitted by the earth’s surface and elsewhere in the atmosphere. These substances emit infrared radiation in all directions, but the net amount emitted to space is typically less than it would have been in the absence of these absorbers because of the decline in temperature with altitude in the troposphere and the consequent weakening of emission. An increase in the concentration of GHGs increases the magnitude of this effect; the difference is sometimes called the enhanced greenhouse effect. The change in a GHG concentration because of anthropogenic emissions contributes to an instantaneous radiative forcing. Surface temperature and troposphere warm in response to this forcing, gradually restoring the radiative balance at the top of the atmosphere.

[viii] Definitions come from the following documents: Health Care Without Harm and ARUP publication: Designing a Net Zero Roadmap for Healthcare[7], Health Care Without Harm Road map[11], Emissions Gap Report 2020[12], IPCC Fifth Assessment Report[14], IPCC Special Report: Global Warming of 1.5 °C[16].

Greenhouse gases (GHG): These are the atmospheric gases responsible for causing global warming and climatic change. The major greenhouse gases are carbon dioxide, methane, and nitrous oxide. Less prevalent but very powerful GHGs are hydrofluorocarbons, perfluorocarbons, and sulphur hexafluoride.

Indirect GHG emissions: These emissions are a consequence of the operations of the institution but occur at sources or institutions owned by another organization or company.

Inventory: This refers to a quantified list of an organization's GHG emissions and sources. In some contexts, it is more commonly known as carbon footprint, which is the term adopted in this tool.

Intergovernmental Panel on Climate Change (IPCC): Created in 1988 by the World Meteorological Organization and the United Nations Environment Programme , the IPCC aims to provide governments at all levels with scientific information to develop climate policies. It provides regular assessments of the scientific basis of climate change, its impacts and future risks, and options for adaptation and mitigation.

Nationally determined contributions (NDC): These are submissions by countries that have ratified the Paris Agreement, which present their national efforts to reach the long-term goal of limiting warming to well below 2°C. New or updated NDCs are to be submitted every five years starting in 2020. NDCs represent a country's current ambition/target for reducing emissions nationally.

Offset (in climate policy): This refers to a unit of CO_2e emissions that is reduced, avoided, or sequestered to compensate for emissions occurring elsewhere.

Paris Agreement: Under the UNFCCC, in December 2015 the Paris Agreement was approved as a legally binding international treaty on climate change with the goal of limiting global warming to well below 2 or preferably 1.5 degrees Celsius, compared to pre-industrial levels.

Scope: This defines operational boundaries in relation to indirect and direct GHG emissions. See table 2 formore information.

United Nations Framework Convention on Climate Change (UNFCCC): The convention was adopted in May 1992 in New York and signed at the 1992 Earth Summit in Rio de Janeiro by more than 150 countries and the European community. Its ultimate objective is the "stabilization of greenhouse gas concentrations in the atmosphere at a level that would prevent dangerous anthropogenic interference with the climate system".
It contains commitments for all parties, including returning greenhouse gas emissions not controlled by the Montreal Protocol to 1990 levels by the year 2000. The convention entered into force in March 1994. In 1997, the UNFCCC adopted the Kyoto Protocol and in 2015, the Paris Agreement.

World Input-Output Database (WIOD): It is an Environmentally Extended Input-Output (EEIO) database developed by the Groningen Growth and Development Centre of the University of Groningen. Input-Output (IO) tables model the economic flows between sectors in an economy. EEIO tables combine this with emissions data to quantify links between economic activity and issues such as resource use, land demand, greenhouse gas emissions, etc. EEIO tables can relate to a single country or region or cover multiple regions with many covering the global economy. EEIO databases cover direct emissions from CO_2 and, in some cases, other GHGs by sector. Through calculations based on EEIO databases, these direct emissions inventories may be used to derive full supply-chain assessments associated with all healthcare system spending.

Zero emissions: It means just that. This is the point where an entity does not produce any carbon equivalent emissions. It should be the ultimate goal of decarbonization. However, most sectors are not likely to reach this point without significant investment, innovation, and technological research.

References

[1] Health Care Without Harm, [Online]. Available: https://noharm.org/.

[2] I. S. J. U. W. a. H. W. Peter-Paul Pichler, International comparison of health care carbon footprints, 2019. Available at: https://iopscience.iop.org/article/10.1088/1748-9326/ab19e1

[3] Health Care Without Harm and ARUP, Health Care's Climate Footprint, 2019. Available at: https://noharm-global.org/climatefootprintreport

[4] The Lancet, The Lancet Countdown - Data platform. Available at: https://www.lancetcountdown.org/data-platform/

[5] World Resources Institute, The Greenhouse Gas Protocol - A Corporate Accounting and Reporting Standard. Available at: https://ghgprotocol.org/corporate-standard

[6] Sulbaek Andersen, Mads P., et al. "Atmospheric chemistry of isoflurane, desflurane, and sevoflurane: kinetics and mechanisms of reactions with chlorine atoms and OH radicals and global warming potentials." The Journal of Physical Chemistry A 116.24 (2012): 5806-5820.

[7] Health Care Without Harm and ARUP publication: Designing a Net Zero Roadmap for Healthcare: Technical Methodology and Guidance.

[8] Intergovernmental Panel on Climate Change, IPCC, 2006 IPCC Guidelines for National Greenhouse Gas Inventories, Japan, 2006. Available at: https://www.ipcc-nggip.iges.or.jp/public/2006gl/

[9] WRI, C40 and ICLEI, Global Protocol for Community-Scale Greenhouse Gas Emission Inventories, 2014. Available at: https://ghgprotocol.org/greenhouse-gas-protocol-accounting-reporting-standard-cities

[10] UNFCCC / Clean Development Mechanism, "Tool to calculate the emission factor for an electricity system. Version 07.0," 2018. [Online]. Available: https://cdm.unfccc.int/methodologies/PAmethodologies/tools/am-tool-07-v7.0.pdf.

[11] Health Care Without Harm and ARUP, Global Road Map for Health Care Decarbonization, 2020. Available at: https://healthcareclimateaction.org/roadmap

[12] UN Environment Programme, Emissions Gap Report 2020, 2020. Available at: https://www.unep.org/emissions-gap-report-2020

[13] Intergovernmental Panel on Climate Change, IPCC, Fourth Assessment Report (AR4), 2007. Available at: https://www.ipcc.ch/assessment-report/ar4/

[14] Intergovernmental Panel on Climate Change, IPCC, Fifth Assessment Report, 2014. Available at: https://www.ipcc.ch/assessment-report/ar5/

[15] Intergovernmental Panel on Climate Change, IPCC, "Special Report - Climate Change and Land," 2019. Available at: https://www.ipcc.ch/srccl/

[16] Intergovernmental Panel on Climate Change, IPCC, «Special Report - Global Warming of 1.5 °C », 2019. Available at: https://www.ipcc.ch/sr15/

Annex I – Global warming potentials

Table 8 – Global warming potentials

Gas or compound	Family/ type	GWP*	Classification	References
CO_2	-	1		1
CH_4	-	25		1
N_2O	-	298	Anesthetic	1
Isoflurane		510	Anesthetic	5
Sevoflurane		130	Anesthetic	5
Desflurane		2,540	Anesthetic	5
HFC-23	HFC	14,800	Refrigerant	1
HFC-32	HFC	675	Refrigerant	1
HFC-41	HFC	92	Refrigerant	1
HFC-125	HFC	3,500	Refrigerant	1
HFC-134	HFC	1,100	Refrigerant	1
HFC-134a	HFC	1,430	Refrigerant	1
HFC-143	HFC	353	Refrigerant	1
HFC-143a	HFC	4,470	Refrigerant	1
HFC-152	HFC	53	Refrigerant	1
HFC-152a	HFC	124	Refrigerant	1
HFC-161	HFC	12	Refrigerant	1
HFC-227ea	HFC	3,220	Refrigerant	1
HFC-236cb	HFC	1,340	Refrigerant	1
HFC-236ea	HFC	1,370	Refrigerant	1
HFC-236fa	HFC	9,810	Refrigerant	1
HFC-245ca	HFC	693	Refrigerant	1
HFC-245fa	HFC	1,030	Refrigerant	1
HFC-365mfc	HFC	794	Refrigerant	1
HFC-43-10mee	HFC	1,640	Refrigerant	1
SF6	-	22,800	Refrigerant	1
NF3	-	17,200	Refrigerant	1
PFC-14	PFC	7,390	Refrigerant	1
PFC-116	PFC	12,200	Refrigerant	1
PFC-218	PFC	8,830	Refrigerant	1

Gas or compound	Family/ type	GWP*	Classification	References
PFC-318	PFC	10,300	Refrigerant	1
PFC-3-1-10	PFC	8,860	Refrigerant	1
PFC-4-1-12	PFC	9,160	Refrigerant	1
PFC-5-1-14	PFC	9,300	Refrigerant	1
PFC-9-1-18	PFC	7,500	Refrigerant	1
Trifluoromethyl Sulfur pentafluoride	PFC	17,700	Refrigerant	1
Perfluorocyclopropane	PFC	17,340	Refrigerant	1
R-11	CFC	4,750	Refrigerant	1
R-12	CFC	10,900	Refrigerant	1; 3
R-22	HCFC	1,810	Refrigerant	1; 3
R-44	-	1	Refrigerant	1
R-123	HCFC	77	Refrigerant	1
R-290	HC	3	Refrigerant	1
R-400	**	0	Refrigerant	1 ; 2
R-401A	**	16	Refrigerant	1 ; 2
R-401B	**	14	Refrigerant	1 ; 2
R-401C	**	19	Refrigerant	1 ; 2
R-402A	**	2,100	Refrigerant	1 ; 2
R-402B	**	1,330	Refrigerant	1 ; 2
R-403A	**	1,766	Refrigerant	1 ; 2
R-403B	**	3,444	Refrigerant	1 ; 2
R-404A	**	3,922	Refrigerant	1 ; 2
R-406A	**	0	Refrigerant	1 ; 2
R-407A	**	2,107	Refrigerant	1 ; 2
R-407B	**	2,804	Refrigerant	1 ; 2
R-407C	**	1,774	Refrigerant	1 ; 2
R-407D	**	1,627	Refrigerant	1 ; 2
R-407E	**	1,552	Refrigerant	1 ; 2
R-407F	**	1,825	Refrigerant	1 ; 2
R-408A	**	2,301	Refrigerant	1 ; 2
R-409A	**	0	Refrigerant	1 ; 2
R-409B	**	0	Refrigerant	1 ; 2
R-410A	**	2,088	Refrigerant	1 ; 2
R-410B	**	2,229	Refrigerant	1 ; 2
R-411A	**	14	Refrigerant	1 ; 2
R-411B	**	4	Refrigerant	1 ; 2

Gas or compound	Family/ type	GWP*	Classification	References
R-412A	**	442	Refrigerant	1 ; 2
R-413A	**	2,053	Refrigerant	1 ; 2
R-414A	**	0	Refrigerant	1 ; 2
R-414B	**	0	Refrigerant	1 ; 2
R-415A	**	22	Refrigerant	1 ; 2
R-415B	**	93	Refrigerant	1 ; 2
R-416A	**	844	Refrigerant	1 ; 2
R-417A	**	2,346	Refrigerant	1 ; 2
R-417B	**	3,027	Refrigerant	1 ; 2
R-417C	**	1,809	Refrigerant	1 ; 2
R-418A	**	3	Refrigerant	1 ; 2
R-419A	**	2,967	Refrigerant	1 ; 2
R-419B	**	2,384	Refrigerant	1 ; 2
R-420A	**	1,258	Refrigerant	1 ; 2
R-421A	**	2,631	Refrigerant	1 ; 2
R-421B	**	3,190	Refrigerant	1 ; 2
R-422A	**	3,143	Refrigerant	1 ; 2
R-422B	**	2,526	Refrigerant	1 ; 2
R-422C	**	3,085	Refrigerant	1 ; 2
R-422D	**	2,725	Refrigerant	1 ; 2
R-422E	**	2,592	Refrigerant	1 ; 2
R-423A	**	2,280	Refrigerant	1 ; 2
R-424A	**	2,440	Refrigerant	1 ; 2
R-425A	**	1,505	Refrigerant	1 ; 2
R-426A	**	1,508	Refrigerant	1 ; 2
R-427A	**	2,138	Refrigerant	1 ; 2
R-428A	**	3,607	Refrigerant	1 ; 2
R-429A	**	12	Refrigerant	1 ; 2
R-430A	**	94	Refrigerant	1 ; 2
R-431A	**	36	Refrigerant	1 ; 2
R-432A	**	0	Refrigerant	1 ; 2
R-433A	**	0	Refrigerant	1 ; 2
R-434A	**	3,245	Refrigerant	1 ; 2
R-435A	**	25	Refrigerant	1 ; 2
R-436A	**	0	Refrigerant	1 ; 2
R-436B	**	0	Refrigerant	1 ; 2
R-437A	**	1,805	Refrigerant	1 ; 2

Gas or compound	Family/ type	GWP*	Classification	References
R-438A	**	2,264	Refrigerant	1 ; 2
R-439A	**	1,983	Refrigerant	1 ; 2
R-440A	**	144	Refrigerant	1 ; 2
R-441A	**	0	Refrigerant	1 ; 2
R-442A	**	1,888	Refrigerant	1 ; 2
R-443A	**	0	Refrigerant	1 ; 2
R-444A	**	87	Refrigerant	1 ; 2
R-445A	**	129	Refrigerant	1 ; 2
R-500	**	32	Refrigerant	1 ; 2
R-501	**	0	Refrigerant	1 ; 2
R-502	**	0	Refrigerant	1 ; 2
R-503	**	5,935	Refrigerant	1 ; 2
R-504	**	325	Refrigerant	1 ; 2
R-505	**	0	Refrigerant	1 ; 2
R-506	**	0	Refrigerant	1 ; 2
R-507 o R-507A	**	3,985	Refrigerant	1 ; 2
R-508A	**	13,214	Refrigerant	1 ; 2
R-508B	**	13,396	Refrigerant	1 ; 2
R-509 o R-509A	**	4,945	Refrigerant	1 ; 2
R-510A	**	0	Refrigerant	1 ; 2
R-511A	**	0	Refrigerant	1 ; 2
R-512A	**	189	Refrigerant	1 ; 2
R-600	HC	4	Refrigerant	6
R-600a	HC	3	Refrigerant	4
R-717		0	Refrigerant	4
R-718		0	Refrigerant	
R-1234YF	HFO	4	Refrigerant	4
R-1234ZE	HFO	7	Refrigerant	4
R-1233ZD	HFCO	1	Refrigerant	4

* GWP values for 100-year time horizon

** Compound of more than one type of refrigerant

Sources:

1: IPCC – Fourth Assessment Report. Table 2.14 (2007). Available at: https://www.ipcc.ch/publications_and_data/ar4/wg1/en/ch2s2-10-2.html

2: ASHRAE - Standard 34 - Designation and Safety Classification of Refrigerants (2010)

3: Green House Gas Protocol - Global Warming Potential Values – Available at: https://www.ghgprotocol.org/sites/default/files/ghgp/Global-Warming-Potential-Values%20%28Feb%2016%202016%29_1.pdf

4: UN Enviroment - GWP-ODP Calculator. Available at: https://www.unenvironment.org/ozonaction/gwp-odp-calculator

5: Sulbaek Andersen, Mads P., et al. "Atmospheric chemistry of isoflurane, desflurane, and sevoflurane: kinetics and mechanisms of reactions with chlorine atoms and OH radicals and global warming potentials." The Journal of Physical Chemistry A 116.24 (2012): 5806-5820

6: IPCC – Fourth Assessment Report. Tabla 2.15 (2007). Available at: https://www.ipcc.ch/publications_and_data/ar4/wg1/en/ch2s2-10-2.html

Annex II – Online platform visualization

Step 1. Log in here

Step 2. Click on the Hippocrates logo

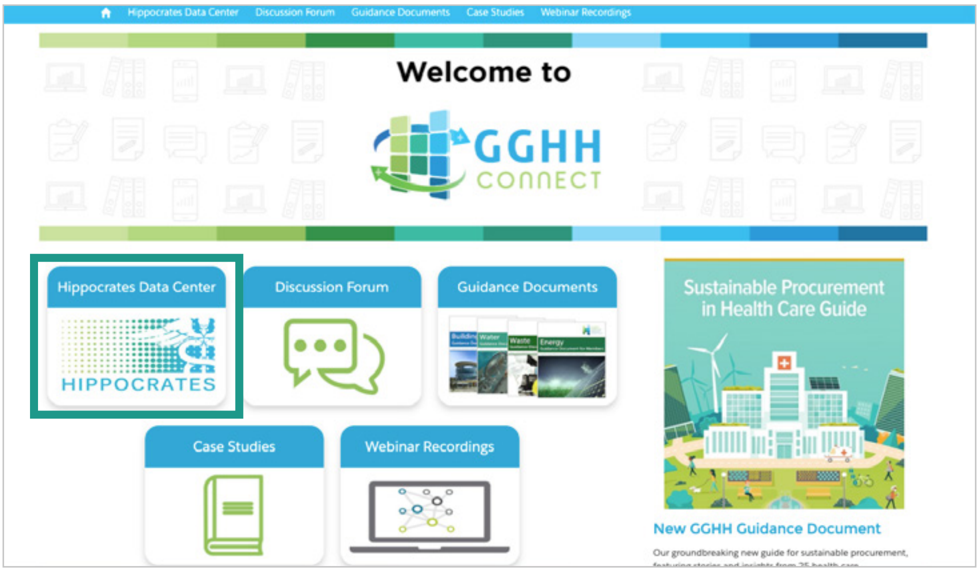

Step 3. Select Data Forms

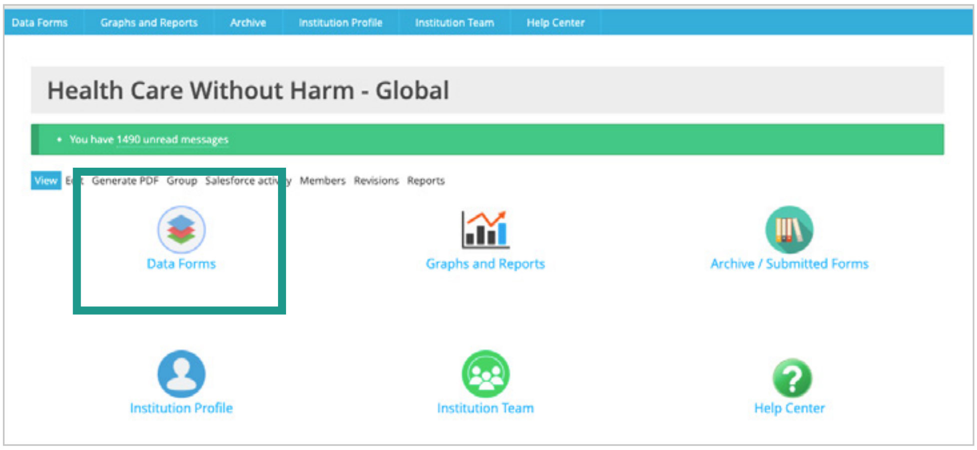

Step 4. Select Climate Impact Checkup or carbon footprint and start a new form

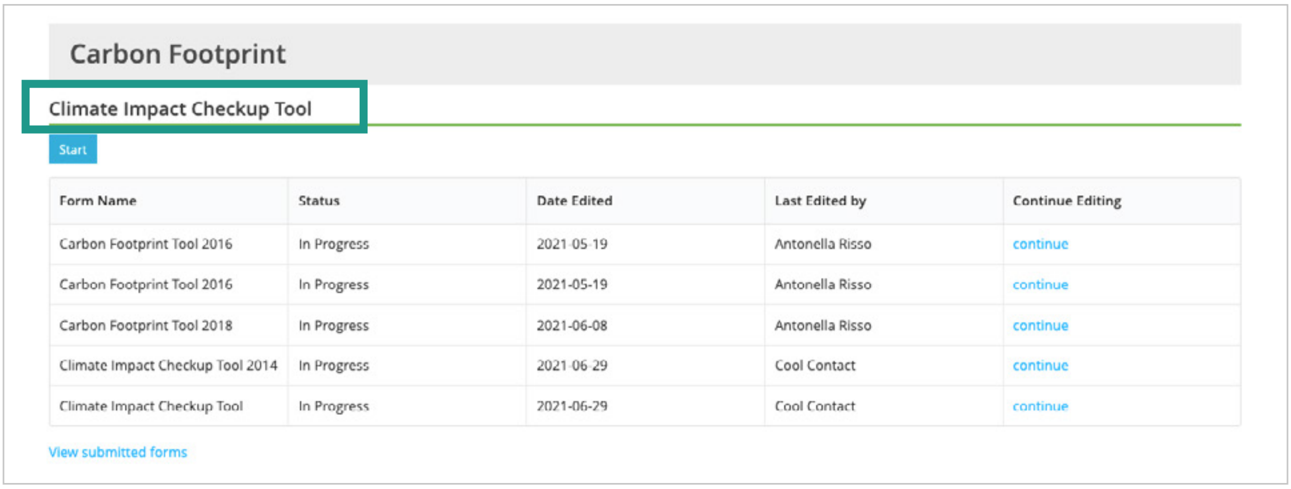

Carbon Footprint

Climate Impact Checkup Tool

Start

Form Name	Status	Date Edited	Last Edited by	Continue Editing
Carbon Footprint Tool 2016	In Progress	2021-05-19	Antonella Risso	continue
Carbon Footprint Tool 2016	In Progress	2021-05-19	Antonella Risso	continue
Carbon Footprint Tool 2018	In Progress	2021-06-08	Antonella Risso	continue
Climate Impact Checkup Tool 2014	In Progress	2021-06-29	Cool Contact	continue
Climate Impact Checkup Tool	In Progress	2021-06-29	Cool Contact	continue

View submitted forms

Step 5. Follow the steps and use the tool by Sections Intro/Scope 1/ Scope 2/ Scope 3/ Waste and Comments. **Read the instructions before saving data**. Remember to **Save** before going to the next section.

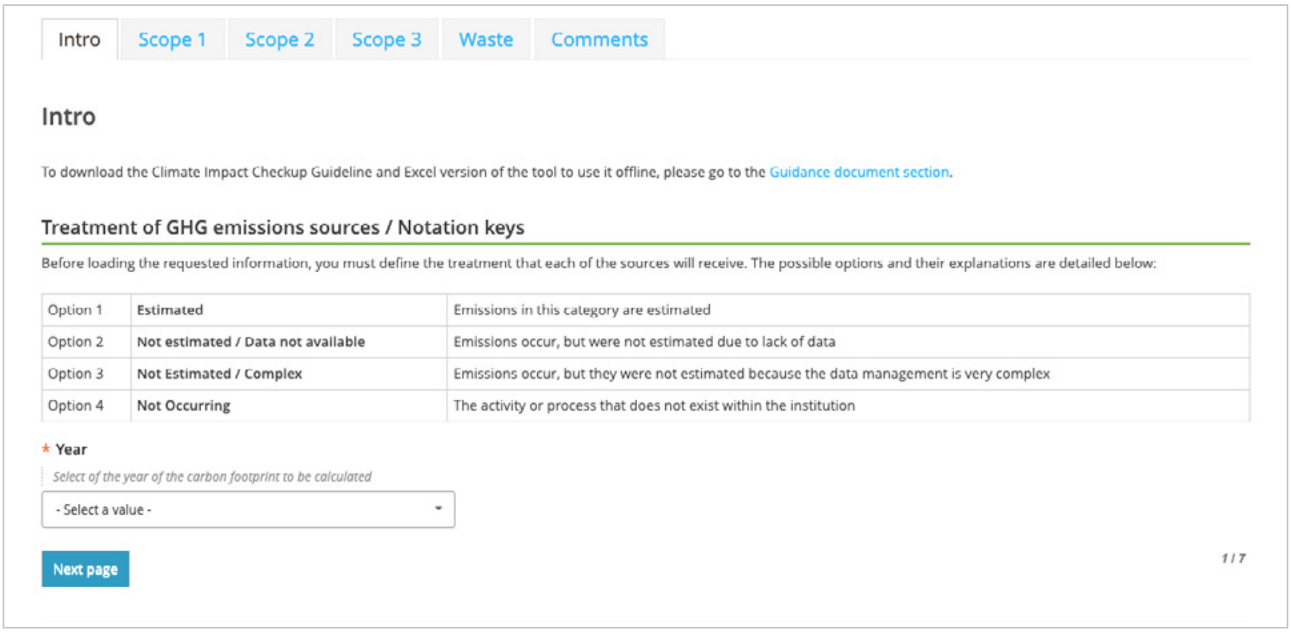

Intro | Scope 1 | Scope 2 | Scope 3 | Waste | Comments

Intro

To download the Climate Impact Checkup Guideline and Excel version of the tool to use it offline, please go to the Guidance document section.

Treatment of GHG emissions sources / Notation keys

Before loading the requested information, you must define the treatment that each of the sources will receive. The possible options and their explanations are detailed below:

Option 1	Estimated	Emissions in this category are estimated
Option 2	Not estimated / Data not available	Emissions occur, but were not estimated due to lack of data
Option 3	Not Estimated / Complex	Emissions occur, but they were not estimated because the data management is very complex
Option 4	Not Occurring	The activity or process that does not exist within the institution

* Year

Select of the year of the carbon footprint to be calculated

- Select a value -

Next page

1 / 7

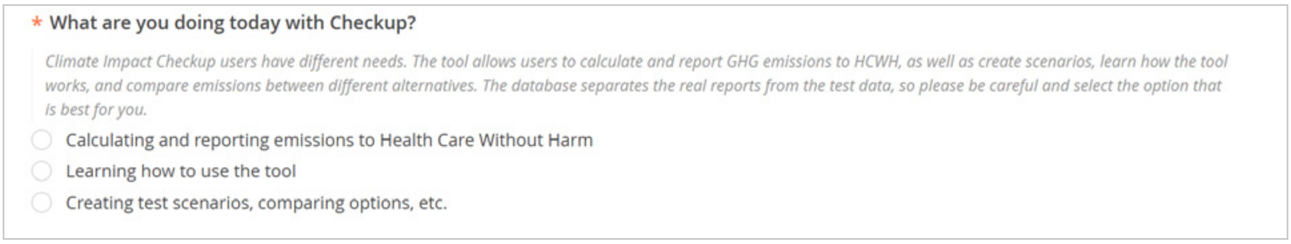

* What are you doing today with Checkup?

Climate Impact Checkup users have different needs. The tool allows users to calculate and report GHG emissions to HCWH, as well as create scenarios, learn how the tool works, and compare emissions between different alternatives. The database separates the real reports from the test data, so please be careful and select the option that is best for you.

- Calculating and reporting emissions to Health Care Without Harm
- Learning how to use the tool
- Creating test scenarios, comparing options, etc.

Intro | Scope 1 | Scope 2 | Scope 3 | Waste | Comments

Scope 1

Scope 1 emissions are those that occur from sources that are owned or controlled by the health care facility. For example: emissions due to combustion in boilers, water heaters or ambulances, the use of Nitrous Oxide as an anesthetic or refrigerant leaks in air conditioning systems.

1.1 Stationary Combustion

In this section the GHG emissions resulting from the burning of fuels in stationary processes are estimated. Stationary combustion is that which occurs in devices that do not move, like boilers, hot water tanks, stoves, water heaters, backup generators, on site waste incineration, autoclaves, etc.

Clarification. CO2 emissions from biofuels (biodiesel, bioethanol, firewood, etc.) are considered neutral in net terms (the amount of CO2 that biomass has captured throughout its life is equal to the amount that it then emits when burned) and that is why they are not counted, but are expressed as zero.

* **Indicate what will be the treatment of this source category**

- (●) Estimated
- ( ) Not Estimated / Data not available
- ( ) Not Estimated / Complex
- ( ) Not Occurring

Instructions
1 - Enter the amount consumed at the institution for each type of fuel in the "Amount consumed" column. If any fuel is not used, leave the respective cell unfilled.
2 - Select the unit for each fuel from the scrolldown menu in the "Unit" column.

			Emissions (kg)				Auxiliar	
Type of fuel	Amount Consumed	Unit	CO_2	CH_4	N_2O	CO_2e	Amount Consumed	Unit
Total								
Natural Gas		- Select -						m3
Liquefied Petroleum Gases (LPG)		- Select -						kg

1.3.2 Medicinal / Anesthetic gases

Unintentional emissions of medicinal and anesthetic gases are included in this section.

* **Indicate what will be the treatment of this source category**

- (●) Estimated
- ( ) Not Estimated / Data not available
- ( ) Not Estimated / Complex
- ( ) Not Occurring

Instructions
1 - For each of the anesthetic gases enter the sizes of the bottles in ml (the tool allows up to three different sizes).
2 - Indicate the number of bottles consumed by gas and size.

Anesthetic Agent	Bottle Size (ml)	Number of Bottles	ml	liters	Density (kg/l)	GWP	Emissions (kgCO2e)
Isoflurane							
Bottle Size 1							
Bottle Size 2							
Bottle Size 3							
Total					1.496	510	
Anesthetic Agent	Bottle Size (ml)	Number of Bottles	ml	liters	Density (kg/l)	GWP	Emissions (kgCO2e)
Sevoflurane							
Bottle Size 1							
Bottle Size 2							
Bottle Size 3							
Total					1.52	130	

Intro | Scope 1 | Scope 2 | Scope 3 | Waste | Comments

Scope 2

Scope 2 emissions are those that result from the generation of electricity purchased by the institution. The electricity purchased is defined as that which is brought in from outside the limits of the health center.
These emissions do not occur physically in the institution, but where the electricity is generated.

2.1 Purchase of electricity

This section estimates the GHG emissions that result from the burning of fuels in thermal power plants for the generation of electricity.

* Indicate what will be the treatment of this source category

- (•) Estimated
- ( ) Not Estimated / Data not available
- ( ) Not Estimated / Complex
- ( ) Not Occurring

Instructions
1 - Enter the amount of electricity consumed (in kWh) in the appropriate column.

	Consumed quantity	Unit	Emissions (kgCO_2e)
Grid electricity		kWh	

Previous page | Next page — 3 / 7

Review and submit form | Save and continue | Save as draft

2.2 Purchased steam, hot or chilled water

In some countries, people can purchase steam, hot or chilled water. In this section, emissions that result from the production of these items are estimated.

* Indicate what will be the treatment of this source category

- (•) Estimated
- ( ) Not Estimated / Data not available
- ( ) Not Estimated / Complex
- ( ) Not Occurring

United States & Canada
1- Enter the amount of purchased steam, hot or chilled water (in kbtu) in the appropriate row and column.

Item	Consumed quantity	Unit	Emissions (kgCO_2e)
Total			
Steam		kbtu	
Hot Water		kbtu	
Chilled Water- Electric Driven Chiller		kbtu	
Chilled Water- Absorption Chiller using Natural Gas		kbtu	
Chilled Water- Engine-Driven Chiller using Natural Gas		kbtu	

Intro | Scope 1 | Scope 2 | Scope 3 | Waste | Comments

Scope 3

Scope 3 emissions result from activities of the institution, but do not occur from controlled or owned sources. For example, emissions due to the transport of workers from their homes to the hospital.

3.1 Business trips

This section estimates the GHG emissions resulting from the burning of fuel in transportation carried out for business trips. For example: attendance at conferences / workshops / seminars, meetings in places outside the hospital, transfer of supplies made by suppliers (if it is possible to get this information), etc.

* Indicate what will be the treatment of this source category

- (•) Estimated
- ( ) Not Estimated / Data not available
- ( ) Not Estimated / Complex
- ( ) Not Occurring

Instructions
1 - In the first column, choose the type of transport used from the drop-down menu.
2 - In the second and third column enter the origin and destination of the trip.
3 - In the fourth column, indicate the distance (in km) of the route that is made. If you have the distance in miles, multiply that value by 1.609 to convert it to km (1 mile = 1.609 km).

Clarifications.
- It is only needed to enter the distance of one way, then the tool will multiply it by two to consider the round trip.
- If there are hospital members who share a car, van, or motorcycle ride, these carpool rides need to be entered only once.
- Only data with with a (*) are needed to estimate emissions. The rest of the requested data will provide you more information on this source to better define mitigation actions afterwards.

Review and submit form | Save and continue | Save as draft

3.2 Employee commuting

This section estimates the GHG emissions that result from the burning of fuel in the transportation carried out by health center workers to and from the institution.

* **Indicate what will be the treatment of this source category**

- (●) Estimated
- ( ) Not Estimated / Data not available
- ( ) Not Estimated / Complex
- ( ) Not Occurring

Instructions

1 - In the first column, choose the type of transport used from the drop-down menu.
2 - In the second column indicate the distance (in km) of the route that is made. If you have the distance in miles, multiply that value by 1.609 to convert it to km (1 mile = 1.609 km).
3 - In the third column indicate the number of days per week that you do this commuting.

Clarifications.

- It is only needed to enter the distance of one way, then the tool will multiply it by two to consider the round trip.
- If there are hospital members who share a car, van, or motorcycle ride, these carpool rides need to be entered only once.

Transport	Distance (km)	Number of days a week (d)	Distance per week (km/sem)	Distance per year(km)
- Select -				
- Select - / Private car (diesel) / Private car (gasoline) / Private car (CNG) / Bus				
- Select -				

Review and submit form | Save and continue | Save as draft

3.3 Patient commuting

This section estimates the GHG emissions that result from the burning of fuel in the transportation carried out by patients from the health center to and from the institution.

* **Indicate what will be the treatment of this source category**

- (●) Estimated
- ( ) Not Estimated / Data not available
- ( ) Not Estimated / Complex
- ( ) Not Occurring

* **Length of the sample period**

- ( ) 7 days
- ( ) 1 month
- ( ) Annual

Instructions

1 - In the first column, choose the type of transport used from the drop-down menu.
2 - In the second column indicate the distance (in km) of the route that is made. If you have the distance in miles, multiply that value by 1.609 to convert it to km [illegible]
3 - In the third column, choose the reason of your visit to the hospital from the drop-down menu.

Clarifications.

- It is only needed to enter the distance of one way, then the tool will multiply it by two to consider the round trip.
- If there are hospital members who share a car, van, or motorcycle ride, these carpool rides need to be entered only once.

Transport	Distance (km)	[illegible]
- Select -		- Select - (- Select - / Medical consultations and outpatient procedures / Internment / Withdrawal from studies or medication)
- Select -		- Select -
[illegible]		[illegible]

Review and submit form | Save and continue | Save as draft

3.4 Inhalers

This section estimates the GHG emissions that result from the use of metered dose inhalers (MDI) and dry powder inhalers (DPI).

* Indicate what will be the treatment of this source category

- (●) Estimated
- ( ) Not Estimated / Data not available
- ( ) Not Estimated / Complex
- ( ) Not Occurring

Instructions

1 - Enter de number of inhalers per type along with the quantity of doses for all the options applicable to your institution (dispensed at the institution, sold/lent in the central pharmacy and/or prescribed by doctors).
2 - For the prescribed inhalers, the percentage of inhalers that are finally bought and not accounted in the previous categories can be modified if you consider that it does not reflect the situation of your institution.

MDI: Metered dose inhaler
DPI: Dry powder inhaler

Dispensed at the institution			
Number of inhalers	Type of inhaler	Quantity of doses per inhaler	Emissions (kgCO2e)
Total	All		
Total	MDI		
Total	DPI		
	- Select -		

Review and submit form | Save and continue | Save as draft

3.5 Extra Supply Chain

* Indicate what will be the treatment of this source category

- (●) Estimated
- ( ) Not Estimated / Data not available
- ( ) Not Estimated / Complex
- ( ) Not Occurring

* Local Currency

- Select a value -

* US Dollar Conversion Rate

Instructions
1 - Select your local currency from the scrolldown menu data.
2 - Enter the USD Conversion rate (how much a dollar is in your country currency). Use the average of the year you are considering
3 - Enter your spend for each category
4 - Make sure that you put in all your non pay spend into the relevant rows (Non pay spend is Total Spend - Wages).
5 - Do the completeness cross-check to ensure that all the expenses, and so the emissions of the extra supply chain, are included.

Clarifications:
- Currency conversion rate can be get from:
* Internal Revenue Service of USA (https://www.irs.gov/individuals/international-taxpayers/yearly-average-currency-exchange-rates)
* OANDA (https://www1.oanda.com/currency/converter/). To estimate the average, use the first and the last day of the year.
- Spend is without Sales/VAT taxes
- Emissions factors per category of spend and nation are available in the sheet called "Supply chain_Emissions factors" in the excel version of the tool.
- More information on the disintegration of each category of spend is available in the sheet called "Supply chain_Mapping of sectors" in the excel version of the tool.
- There is information on Multi Regional Input/Output models and WIOD dataset in the refererences section.
- WIOD dataset covers 43 nations. When there is not specific emission factors for a given nation, the values of the group called "Rest of the world" are applied.

Intro | Scope 1 | Scope 2 | Scope 3 | Waste | Comments

Waste

In this section the emissions of management, treatment and disposal of waste produced in the hospital are estimated no matter these activities take place in situ or outside the facility.

Composition of the non-hazardous / general health care waste

Instructions
If you know the composition (in percentage) of the non-hazardous / general health care waste produced in your institution, choose the option "Yes" and enter it in the corresponding cells. Otherwise, choose the option "No" so that the default values for your country are adopted.

* Do you know the composition of your non-hazardous / general health care waste? In percentage (%)

- ( ) Yes
- (●) No

Solid waste disposal

In this section, methane (CH_4) emissions that occur in anaerobic processes that take place in solid waste disposal sites (landfills or garbage dumps) are estimated.

* Indicate what will be the treatment of this source category

- (●) Estimated
- ( ) Not Estimated / Data not available
- ( ) Not Estimated / Complex
- ( ) Not Occurring

Review and submit form | Save and continue | Save as draft

Intro | Scope 1 | Scope 2 | Scope 3 | Waste | Comments

Waste

In this section the emissions of management, treatment and disposal of waste produced in the hospital are estimated no matter these activities take place in situ or outside the facility.

Composition of the non-hazardous / general health care waste

Instructions
If you know the composition (in percentage) of the non-hazardous / general health care waste produced in your institution, choose the option "Yes" and enter it in the corresponding cells. Otherwise, choose the option "No" so that the default values for your country are adopted.

* **Do you know the composition of your non-hazardous / general health care waste? In percentage (%)**

- (●) Yes
- ( ) No

	Composition of your municipal solid waste in percentage (%)
Paper / cardboard	
Textiles	
Food waste	
Wood	
Garden and Park waste	

Review and submit form | Save and continue | Save as draft

Incineration

In this section, the Carbon Dioxide (CO2) emissions that result from the combustion of the fossil carbon fraction of solid waste are estimated. Carbon Dioxide (CO2) emissions from the organic carbon fraction are considered biogenic (neutral in net terms) and are therefore not accounted for.

* **Indicate what will be the treatment of this source category**

- (●) Estimated
- ( ) Not Estimated / Data not available
- ( ) Not Estimated / Complex
- ( ) Not Occurring

* **Does this activity take place in situ or ex situ of the facility?***

** In situ activities are accounted under the Scope 1 while Ex situ activities under Scope 3.*

- ( ) In situ
- (●) Ex situ

Instructions
1 - Enter the amount of each type of waste that it is incinerated.
2 - Select the unit from the scrolldown menu in the "Unit" column.

Type	Amount of waste being incinerated	Unit	Moisture content of hospital waste	Total carbon content in% of dry weight	Fossil carbon fraction in % of total carbon content	Oxidation factor in % of carbon input	Emissions (kgCO2e)	Amount of waste being incinerated	Unit
Total									
Municipal solid waste		- Select -							kg

Step 6. Add your final comments, review and submit your annual Checkup

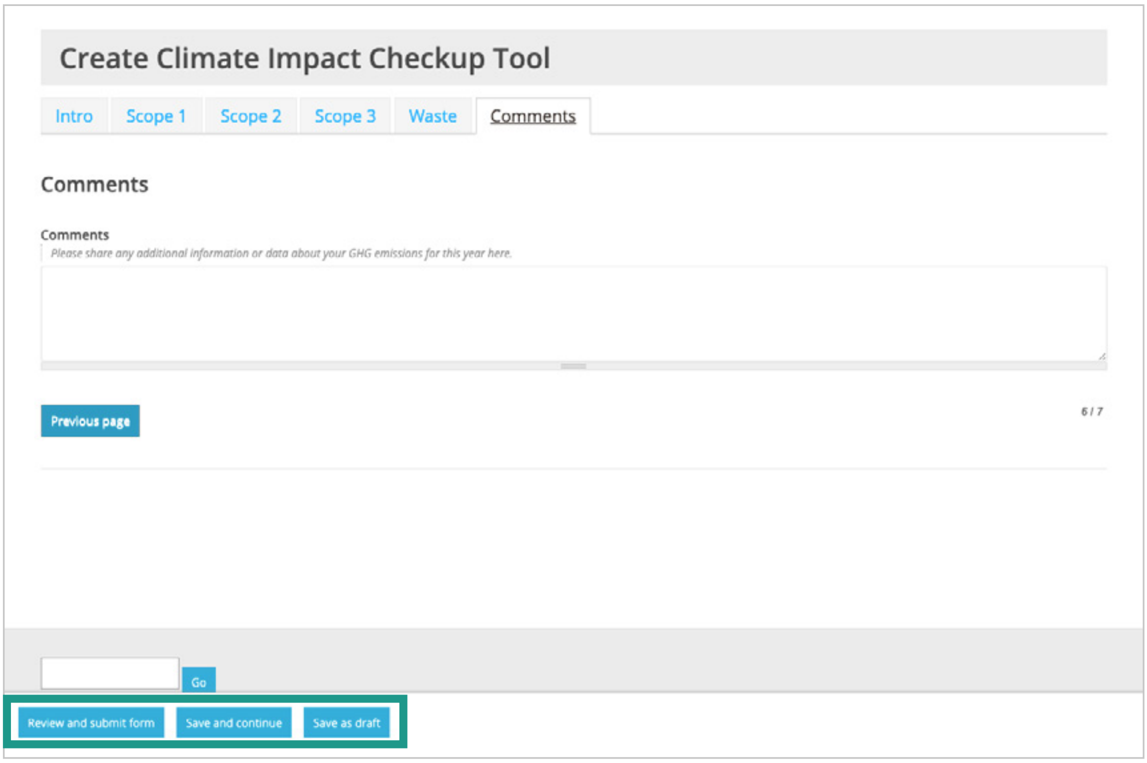

Step 7. Go to the Results page to visualize the graphics, global map, and benchmark your results

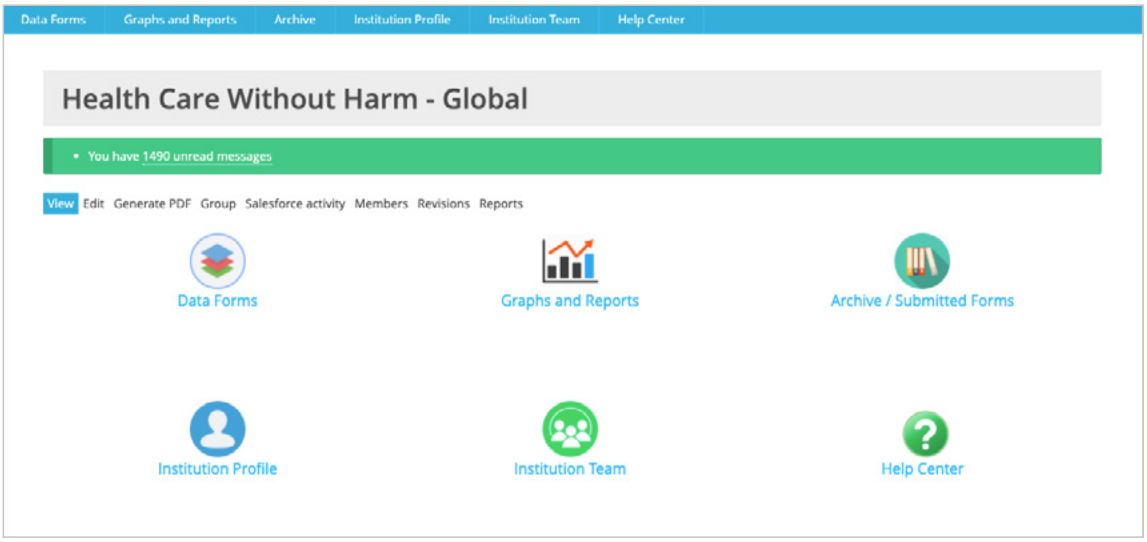

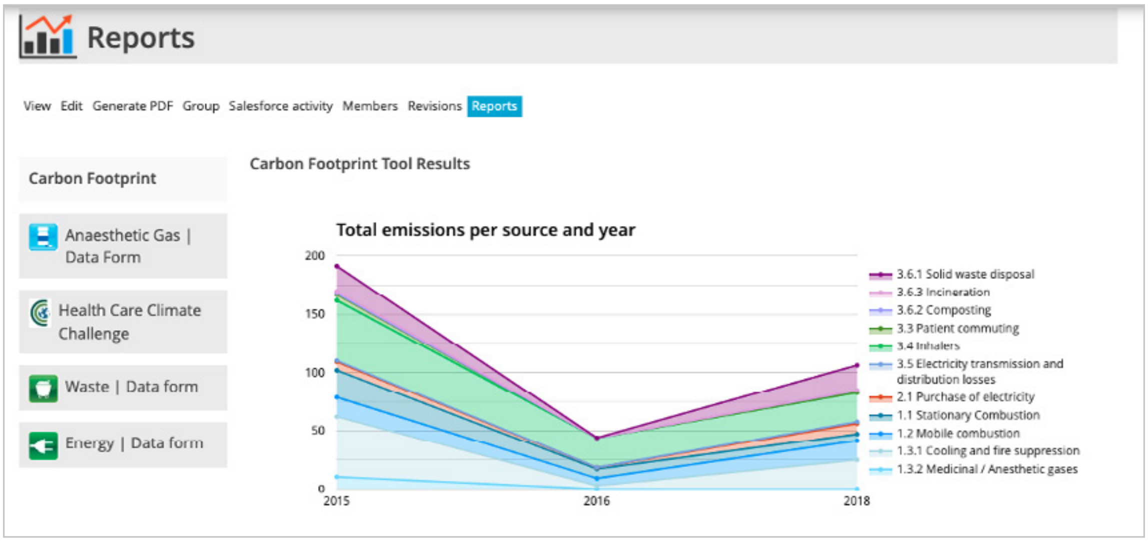

Step 8. Check your Archive

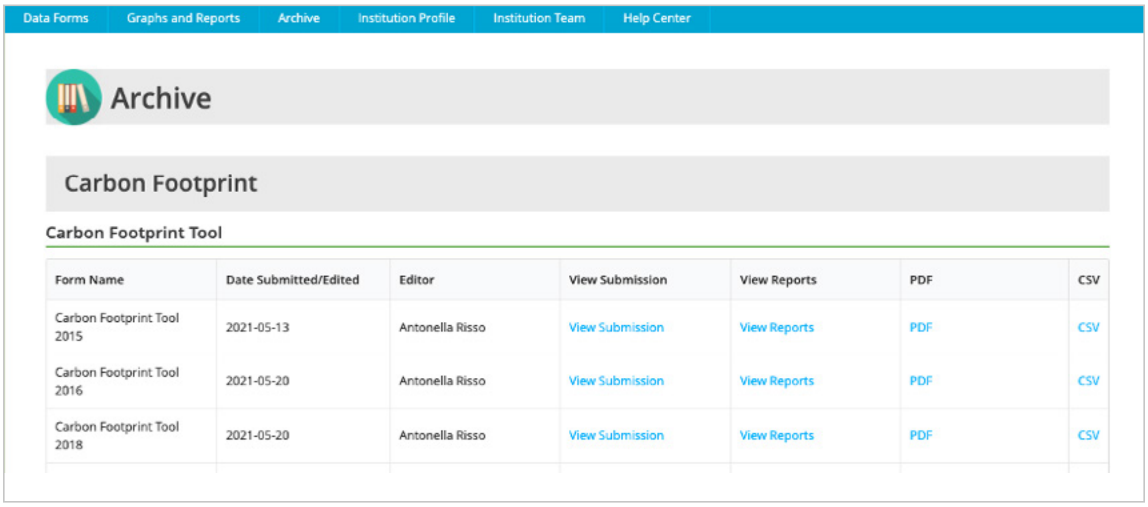

Form Name	Date Submitted/Edited	Editor	View Submission	View Reports	PDF	CSV
Carbon Footprint Tool 2015	2021-05-13	Antonella Risso	View Submission	View Reports	PDF	CSV
Carbon Footprint Tool 2016	2021-05-20	Antonella Risso	View Submission	View Reports	PDF	CSV
Carbon Footprint Tool 2018	2021-05-20	Antonella Risso	View Submission	View Reports	PDF	CSV

Annex III – WIOD categories

Table 9 – Grouped categories, definition and WIOD subcategories for the Extra Supply Chain estimation.

Grouped sectors (Category)	WIOD definition	WIOD subcategories (Health usage)
Business services	Emissions associated with professional services procured by the health sector, like legal, accountancy, and consultancy services	Warehousing and support activities for transportation
		Postal and courier activities
		Financial service activities, except insurance and pension funding
		Insurance, reinsurance, and pension funding, except compulsory social security
		Activities auxiliary to financial services and insurance activities
		Real estate activities
		Legal and accounting activities, activities of head offices, and management consultancy activities
		Scientific research and development
		Advertising and market research
		Other professional, scientific, technical, and veterinary activities
		Administrative and support service activities
		Public administration and defence and compulsory social security
		Education
		Other service activities
		Activities of households as employers, undifferentiated goods- and services producing activities of households for own use
		Architectural and engineering activities, technical testing, and analysis
		Activities of extraterritorial organisations and bodies
		Human health and social work activities

Grouped sectors (Category)	WIOD definition	WIOD subcategories (Health usage)
Construction	Emissions associated with the construction of buildings and infrastructure, including the supply and manufacture of construction materials	Construction<br>Repair and installation of machinery and equipment<br>Manufacture of other non-metallic mineral products<br>Manufacture of basic metals<br>Manufacture of wood and of products of wood and cork, except furniture, and manufacture of articles of straw and plaiting materials<br>Manufacture of fabricated metal products, except machinery and equipment
Food and Catering	Emissions associated with the food products and catering services provided by the health system and accommodation required by health workers	Crop and animal production, hunting, and related service activities<br>Fishing and aquaculture<br>Manufacture of food products, beverages, and tobacco products<br>Accommodation and food service activities
Information and communication technologies	Emissions associated with IT and communication services procured by the health sector, including computer systems, telecoms, and publishing activities	Publishing activities<br>Motion picture, video, and television program production, sound recording and music publishing activities, and programming and broadcasting activities<br>Telecommunications<br>Computer programming, consultancy, and related activities, information service activities
Manufactured fuels, chemicals, and gases	Emissions associated with the production of purchased chemicals, like soap and detergents, and gases used in the health setting	Manufacture of chemicals and chemical products
Medical instruments/ equipment	Emissions associated with purchased medical instruments and equipment, including computers, electronics, and optical products	Mining and quarrying

Grouped sectors (Category)	WIOD definition	WIOD subcategories (Health usage)
Other manufactured products	Emissions associated with purchased products including plastics, textiles, machinery, vehicles, and electrical equipment	Manufacture of computer, electronic, and optical products
		Manufacture of furniture and other manufacturing
		Forestry and logging
		Manufacture of textiles, wearing apparel, and leather products
		Manufacture of rubber and plastic products
		Manufacture of motor vehicles, trailers, and semi-trailers
		Manufacture of other transport equipment
		Manufacture of machinery and equipment not elsewhere classified (n.e.c.).
		Manufacture of electrical equipment
		Retail trade, except of motor vehicles and motorcycles
Paper products	Emissions associated with the production of paper and cardboard products procured by the health sector	Manufacture of paper and paper products
		Printing and reproduction of recorded media
Pharmaceuticals	Emissions associated with the production of pharmaceuticals procured by the health sector, encompassing the emissions associated with the energy, materials, and transportation of pharmaceuticals	Manufacture of basic pharmaceutical products and pharmaceutical preparations
Water and sanitation	Emissions associated with water collection, treatment, supply, and sewerage	Water collection, treatment, and supply
Other procurement	Emissions associated with goods purchased in bulk through wholesalers and intermediaries	Wholesale and retail trade and repair of motor vehicles and motorcycles
		Wholesale trade, except of motor vehicles and motorcycles

Note: WIOD data was grouped in spend categories. The 11 categories shown in this table are the ones used in the Climate Impact Checkup tool to estimate GHG emissions of the extra supply chain. The remaining four categories are electricity, fossil fuels (coal and oil), waste products and recycling, and transport. These were removed to avoid overlaps and double-counting, and are the ones considered in the completeness cross-check section.

Source: HCWH and ARUP. Global Road Map for Health Care Decarbonization (2020)[11]

Annex A: Technical Report, Table A.4 and Table A.5. 2021.

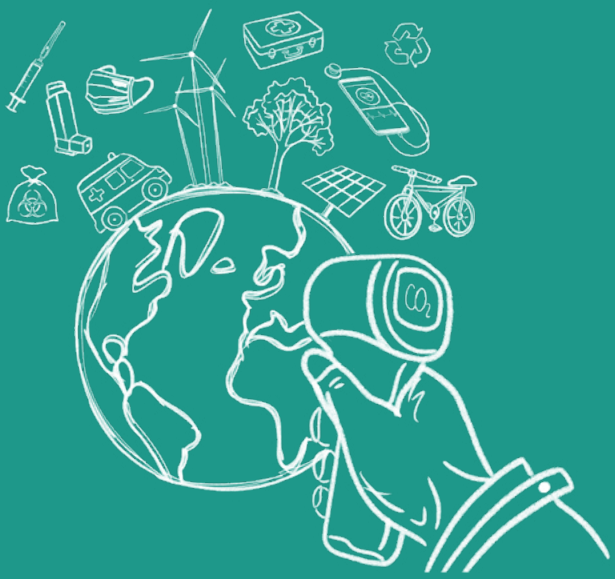

Health Care Without Harm Asia

Unit 203, Kalayaan Center Building
65 V Luna Road cor. Kalayaan AvenueBrgy.
Pinyahan
Quezon City 1101, Philippines
e: info@no-harm.org
www.noharm-asia.org

Health Care Without Harm Europe

Rue de la Pépinière B1000
Brussels, Belgium
e: europe@hcwh.org
www.noharm-europe.org

Health Care Without Harm United States and Canada

12110 Sunset Hills Road
Suite 600
Reston, VA 20190
United States
ph: +1 703 860 9790
fax: +1 703 860 9795
e: info@hcwh.org
www.noharm-uscanada.org

Health Care Without Harm Latin America

e: info@saludsindano.org
www.saludsindano.org

GLOBAL GREEN and HEALTHY HOSPITALS

Health Care Without Harm
